# Study of *VIPER* and *TATE* in kinetoplastids and the evolution of tyrosine recombinase retrotransposons

**DOI:** 10.1186/s13100-019-0175-2

**Published:** 2019-08-05

**Authors:** Yasmin Carla Ribeiro, Lizandra Jaqueline Robe, Danila Syriani Veluza, Cyndia Mara Bezerra dos Santos, Ana Luisa Kalb Lopes, Marco Aurélio Krieger, Adriana Ludwig

**Affiliations:** 1 Pós-Graduação em Biologia Celular e Molecular, Universidade Federal do Paraná, Curitiba, PR Brazil; 2 Departamento de Ecologia e Evolução, Universidade Federal de Santa Maria, Santa Maria, RS Brazil; 3 Curso de Biologia, Universidade Federal do Paraná, Curitiba, PR Brazil; 4 Instituto Carlos Chagas, Fundação Oswaldo Cruz-Fiocruz, Curitiba, PR Brazil

**Keywords:** Tyrosine recombinase, DIRS, Transposable elements, Phage integrase, Hepadnavirus, Retroelements

## Abstract

**Background:**

Kinetoplastids are a flagellated group of protists, including some parasites, such as *Trypanosoma* and *Leishmania* species, that can cause diseases in humans and other animals. The genomes of these species enclose a fraction of retrotransposons including *VIPER* and *TATE*, two poorly studied transposable elements that encode a tyrosine recombinase (YR) and were previously classified as DIRS elements. This study investigated the distribution and evolution of *VIPER* and *TATE* in kinetoplastids to understand the relationships of these elements with other retrotransposons.

**Results:**

We observed that *VIPER* and *TATE* have a discontinuous distribution among Trypanosomatidae, with several events of loss and degeneration occurring during a vertical transfer evolution. We were able to identify the terminal repeats of these elements for the first time, and we showed that these elements are potentially active in some species, including *T. cruzi* copies of *VIPER*. We found that *VIPER* and *TATE* are strictly related elements, which were named in this study as *VIPER-like*. The reverse transcriptase (RT) tree presented a low resolution, and the origin and relationships among YR groups remain uncertain. Conversely, for RH, *VIPER-like* grouped with *Hepadnavirus*, whereas for YR, *VIPER-like* sequences constituted two different clades that are closely allied to *Crypton*. Distinct topologies among RT, RH and YR trees suggest ancient rearrangements/exchanges in domains and a modular pattern of evolution with putative independent origins for each ORF.

**Conclusions:**

Due to the presence of both elements in *Bodo saltans,* a nontrypanosomatid species, we suggested that *VIPER* and *TATE* have survived and remained active for more than 400 million years or were reactivated during the evolution of the host species. We did not find clear evidence of independent origins of *VIPER-like* from the other YR retroelements, supporting the maintenance of the DIRS group of retrotransposons. Nevertheless, according to phylogenetic findings and sequence structure obtained by this study and other works, we proposed separating DIRS elements into four subgroups: *DIRS-like, PAT-like, Ngaro-like,* and *VIPER-like.*

**Electronic supplementary material:**

The online version of this article (10.1186/s13100-019-0175-2) contains supplementary material, which is available to authorized users.

## Background

Trypanosomatid species are among the most ancient eukaryotes, comprising more than ten genera that include both monoxenous insect parasites and dixenous species, which alternate between insects and vertebrates (or plants) [1–3]. Species of *Trypanosoma* and *Leishmania* cause some human pathologies, such as Chagas disease (*T. cruzi*), sleeping sickness (*T. brucei*) and leishmaniasis (genus *Leishmania*) and are transmitted by invertebrate vectors [4]. In addition to the importance of these species as pathogens, they have several unique features. The protein-coding genes of trypanosomatids are transcribed polycistronically, and the mature monocistronic mRNAs are generated by a *trans*-splicing mechanism and polyadenylation [5]. Moreover, gene expression in these species is mostly regulated posttranscriptionally, since all polycistronic precursor RNAs seem to be transcribed at approximately the same rate [6].

More than 10 years ago, the reference genome sequences of three trypanosomatids, *T. brucei, T. cruzi* and *L. major,* were published [7–9]. These genomes are highly repetitive (over 50% in *T. cruzi*), mainly due to large gene families of surface molecules, subtelomeric repeats and transposable elements (TEs) (exclusively retrotransposons) [8]. Recently, Pita et al. (2019) compared the repetitive DNA portions among these three genomes using genome-wide, low-coverage Illumina sequencing. These authors estimated that the genome fraction corresponding to retrotransposons ranges from 12.6% in *T. cruzi* to 5.7% in *T. brucei* and only 1.6% in *L. major* [10].

Retrotransposons are eukaryotic mobile elements that move through an RNA intermediate. They constitute an important source of genetic variation and have actively shaped the structure, function, and evolution of genomes [11–14]. Although considerable attention has been devoted to the two major groups of retrotransposons (LTR and non-LTR elements [15, 16]), recent results have confirmed the presence of another ubiquitous group of retroelements [17–21] that encode a tyrosine recombinase (YR) gene instead of integrase (IN) or endonuclease (EN). Wicker et al. (2007) classified the YR-containing retroelements in the order DIRS, named based on the first described YR retrotransposon, the *DIRS1* from the slime mold *Dictyostelium discoideum* [22].

YR elements, in general, contain three long ORFs (open reading frames). ORF1 was considered a putative *gag-like* gene, given the similarities in size and position with the upstream region of LTR retrotransposons. These elements also have ORFs encoding a YR and a reverse transcriptase/RNase H (RT/RH) that are frequently extensively overlapped [23]. In some DIRS elements, it is also possible to find a coding region for a methyltransferase (MT) or a hydrolase whose functions are still unknown. These elements present a diversified structure of repeats that can be primarily of two types: (1) terminal inverted repeats (ITRs) and an internal complementary region (ICR) that is complementary to the beginning of the left ITR and to the end of the right ITR or (2) “split” direct repeats [24].

Wicker et al. (2007) divided the DIRS order into three subfamilies, *DIRS*, *Ngaro,* and *VIPER*. Recently, Poulter and Butler (2015) divided the YR retrotransposons into three major subgroups: *DIRS*, *PAT-like,* and *Ngaro* elements. This subdivision was based on a phylogenetic tree of the RT/RH domains and on structural differences, which led to the separation of *PAT-like* from the *DIRS* subgroup. *VIPER* (*vestigial interposed retroelement*) sequences were not included in the latter analyses. This retroelement was encountered in *T. cruzi* [25], *T. brucei* and *T. vivax* [26] and was considered an extinct retrotransposon due to the absence of potentially functional copies and to the apparent lack of conserved terminal repeat regions [26, 27]. Moreover, there is another element, *TATE* (*telomere associated transposable element*), that could be classified as DIRS and was found in the genome of *L. braziliensis* as *in tandem* clusters inserted at the same relative position within the telomeric hexamer repeats (GGG↑TTA) [28].

Little is known about *VIPER* and *TATE* in trypanosomatids, and several questions about their structure, functioning and origin remain unanswered. In this study, we investigated the distribution of these elements in the available trypanosomatid genomes and provided evidence that some of their copies could still be active in some species. We also performed an evolutionary analysis of retrotransposon RT, RH and YR proteins. Although we failed to fully elucidate the relationships among YR elements, some differences in topologies were recovered for distinct domains, suggesting the occurrence of a modular pattern of evolution with ancient changes in domains. Moreover, our results suggested a close relationship between *VIPER-like* sequences and the hepadnavirus RH region that may reflect a shared origin of this domain. This paper represents a first step in characterizing the functioning and impact of YR-containing TEs in kinetoplastids.

We used the retrotransposon group nomenclature according to the classification system of Wicker et al. (2007) [29]: order LINE; superfamilies *Copia, Gypsy, Bel-Pao,* and *Retrovirus* from order LTR; superfamilies within order DIRS were updated to *DIRS-like*, *PAT-like*, *Ngaro-like,* and *VIPER-like*. The non-italicized term “DIRS” was used to refer to all the YR-containing retrotransposons (order DIRS).

## Results

### *VIPER* and *TATE* present a discontinuous distribution in trypanosomatid species

A total of 44 trypanosomatid genomes were investigated in this study for the presence of *VIPER* and *TATE* together with the genome of *Bodo saltans*, a free-living kinetoplastid species from the Bodonidae family. Almost all analyzed species presented significant tblastn hits for at least one protein of *VIPER* or *TATE* (Additional file 1). Only *Leishmania tarentolae*, *Leptomonas seymouri, Lotmaria passim, Phytomonas sp.* isolates EM1 and HART1 and *Trypanosoma rangeli* did not present any significant hit, suggesting the complete loss of these retrotransposons.

For most species, *VIPER* and *TATE* seem to have degenerated, as seen in Additional file 2; nevertheless, we were not able to evaluate the conservation of several copies located in the extremities of contigs/scaffolds or missing data regions. Moreover, it is important to consider that the definition of the *gag-like* ORF was mostly based on positional information, since this analysis revealed a highly divergent ORF that did not present significant signs for specific domains in most of the evaluated species.

**Fig. 1:**
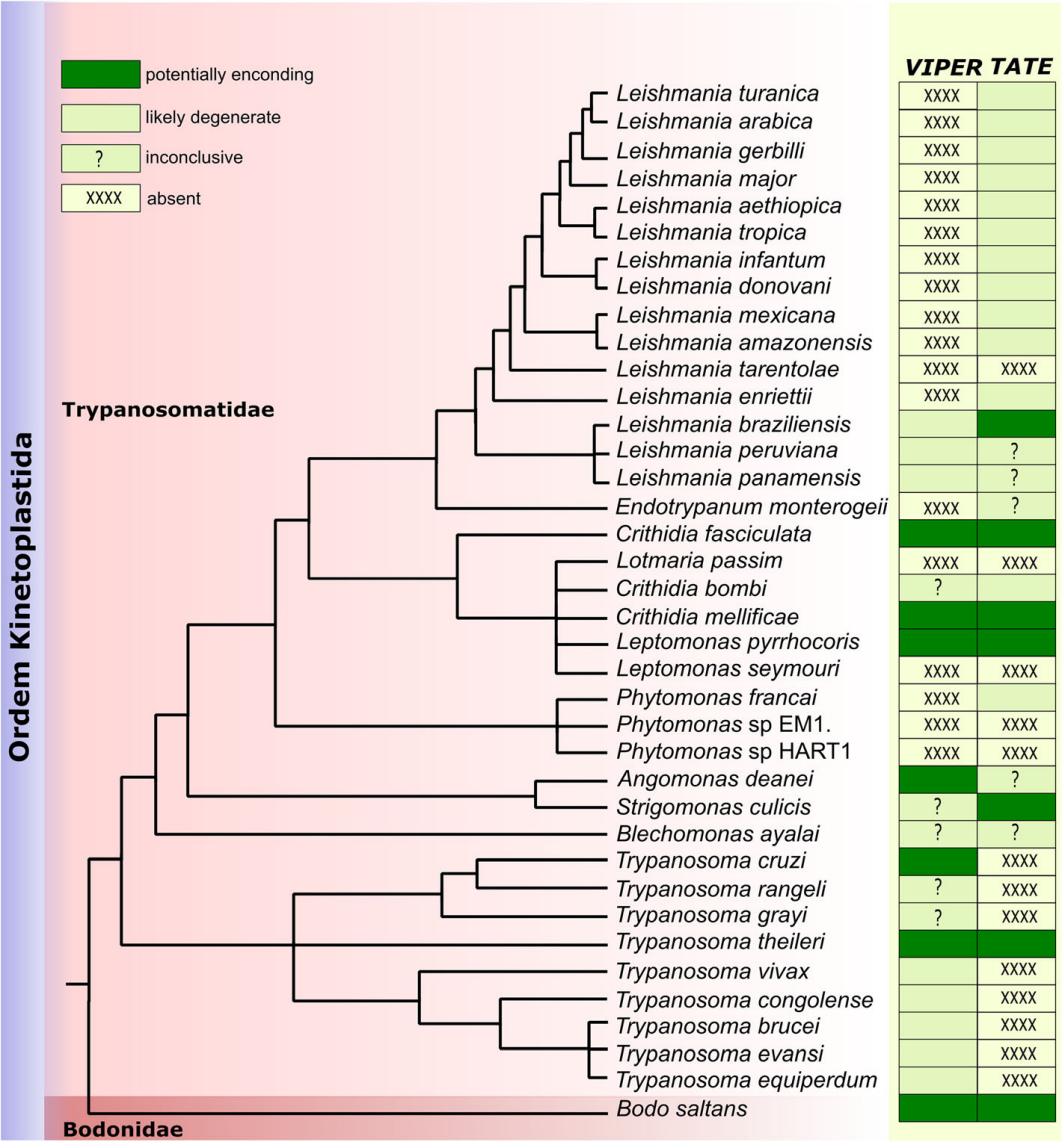
Distribution and conservation of *VIPER* and *TATE* among trypanosomatids. The tree was drawn based on several studies (see text). Branches are not drawn to scale. The presence of at least one potentially encoding copy of the element in the species is indicated as a dark green rectangle. The presence of only degenerate copies is represented by a light green rectangle. Species, where it was not possible to conclude on the conservation of the element, have a question mark, and XXXX represents the probable complete absence of the element

When the status of the copies (potentially encoding, likely degenerate, absent or inconclusive) was summarized on the topology of the trypanosomatid species tree [based on previous studies [1, 30–39]) (Fig. 1), we could observe a patchy distribution of elements among species. The presence of *VIPER* and *TATE* in *B. saltans*, representing the outgroup of the tree, suggests that both TEs were already present in the last common ancestor of this species and trypanosomatids, which diverged more than 460 million years ago (mya) [1]. This ancient existence makes the study of the conservation of these elements throughout trypanosomatid evolution even more exciting.

### *VIPER* is a potentially encoding retrotransposon in some Trypanosomatidae species

*VIPER* seems to have been completely lost in the majority of *Leishmania* species, *Endotrypanum monterogeii*, *Lo. passim*, *Lep*. *seymouri*, *Phytomonas sp*. isolate EM1 and HART1, and *P. francai*. Several other species presented degenerate copies of *VIPER*. In *L. braziliensis, L. panamensis* and *L. peruviana*, which are three closely related species [40, 41], one of the significant hits corresponds to a *VIPER*-derived gene that was already reported [42].

We found at least one potentially encoding copy of *VIPER* in *B. saltans, T. theileri*, *Angomonas deanei*, *Crithidia fasciculata*, *C. mellificae,* and *Lep. pyrrhocoris*. Table 1 and Additional file 3 show a summary of these results. In *T. cruzi*, from four different sequenced genome assemblies, only the *Dm*28c strain presented putative complete encoding copies.

**Table 1:** Summary of *VIPER* copy analyzes from the distinct genomes presenting at least one potentially encoding copy

Species	N^o^ of analyzed copies	N^o^ of copies with at least one potentially encoding ORF	N^o^ of copies with the three potentially encoding ORFs	N^o^ of potential encoding copies with YR and RT/RT related domains	N^o^ of copies with SDRs	Structure of SDRs size in base pairs, percentage of identity	N^o^ of putative autonomous copies^a^	Mean identity among copies in nu (total) and aa levels (for each ORF)^b^
*B. saltans*	10	7	4	1	1^c^	5′ → A1 3′ → B1 → B2 → A2 A: 83 bp, 100%; B: 57 bp, 100%	0	nu: na Gag-like: na YR (5, 150 aa): 32.7% RT/RH (7, 294 aa): 33%
*T. cruzi Dm28c*	15	15	12	12	7	5′ → A1 3′ → B1 → A2 → B2 A: 173 bp, 100%; B: 219 bp, 100%	7	nu (13): 95% Gag-like (13, 492 aa): 97.9% YR (15, 338 aa): 98.2% RT/RH (14, 948 aa): 96.8%
*T. theileri*	15	5	3	2	1 (incomplete)	5′ absent 3′ → A1 → A2 A: 95 bp, 100%;	0	nu: na Gag-like (3, 240 aa): 31.1% YR (2, 272 aa): 47.1% RT/RH (5, 701 aa): 57.6%
*C. fasciculata*	15	7	4	1	3	5′ → A1 3′ → B1 → A2 → B2 1: (A: 136 bp, 99%; B: 288 bp, 100%) 2: (A: 144 bp, 100%; B: 308 bp, 100%) 3: (A: 414 bp, 93%; B: 294 bp, 99%)	1	nu: na Gag -like (3, 240 aa): 31.1% YR (5, 278 aa): 41.6% RT/RH (5, 701 aa): 40.8%
*C. mellificae*	15	4	1	0	? all copies in short or end of contigs	na	0	nu: na Gag-like: na YR (3, 507 aa): 100% RT/RH (2, 393 aa): 43%
*A. deanei*	4	4	1	0	0	na	0	nu: na Gag-like (3, 240 aa): 31.1% YR (2, 272 aa): 48.4% RT/RH (6, 611 aa): 26.7%
*Lep. pyrrhocoris*	15	14	2	0	0	na	0	nu: na Gag-like (2, 396 aa): 99.7% YR (13, 430 aa): 61.6% RT/RH (11, 1091 aa): 72.1%

^a^putative autonomous copies are those containing the tree expected ORFs plus direct repeats

^b^identity was estimated only for regions with confident alignment. The number of sequences and the size of alignment is shown in parenthesis

^c^the pattern of repeats is distinct than expected and the distance from the end of the ORF and the next repeat is quite large and unexpected. Thus, it is not possible to state whether this repetition is indeed part of the element

For most species, the nucleotide alignment of *VIPER* copies was impaired due to the high divergence, indicating that the events of mobilization separating these copies occurred a long time ago. The difference is also high at the amino acid level. In contrast, in *T. cruzi Dm*28c, the nucleotide alignment from conserved copies enabled the identification of the boundaries of the element and the isolation of complete copies that show only 5% mean divergence.

**Fig. 2:**
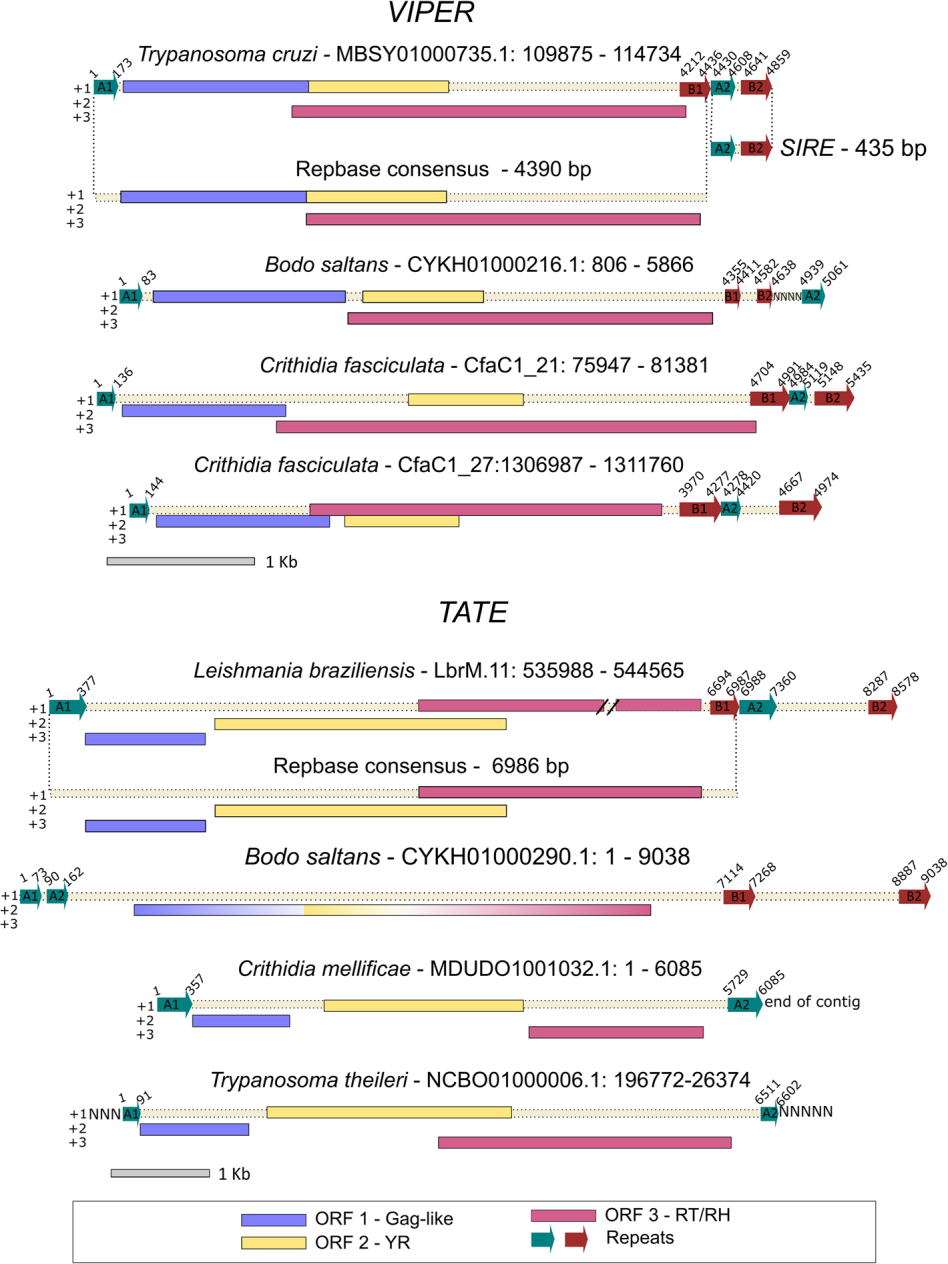
Schematic structure of the potentially complete *VIPER* and *TATE* retroelements found in trypanosomatids. Repbase consensus sequences and the *SIRE* element were also represented. Rectangles of different colors represent the ORFs encoding G*ag-like* (blue), YR (yellow), and RT / RH (pink), whose position was drawn according to the reading phase (+ 1, + 2 or + 3). The arrows indicate the repeats. The scaffold and position of copies are displayed next to the species name. The figure is drawn near to scale (except for some repeats), which is distinct for *VIPER* and *TATE*. The represented *L. braziliensis TATE* copy possesses a frameshift in the third ORF represented as two dashes. NNNN represents missing data regions

We observed that the *VIPER* copies of *T. cruzi* and *C. fasciculata* presented split direct repeats (SDRs) with a pattern similar to that found for *Ngaro* and *PAT-like* elements [18] ordered as A1 at the 5′ end and B1, A2 and B2 at the 3′ end (Fig. 2). For some copies, the related repeats present 100% identity. The 3′ repeats were not present in the Repbase *T. cruzi VIPER* consensus sequence, whereas the 5′ repeat was absent in *T. theileri*. One copy of *B. saltans* presented putative SDRs with a distinct structure of repeats ordered as A1 at the 5′ end and B1, B2, A2 at the 3′ end. For *A. deanei,* it was not possible to evaluate the presence of repeats due to the small size of the contig.

Vasquez et al. (1999, 2000) described the existence of a truncated non-autonomous version of *VIPER*, called *SIRE* (*short interspersed repetitive element*), which was defined by the first 185 bp of *VIPER* 5′ portion, an exclusive internal region of approximately 30 bp, and 220 bp of the *VIPER* 3′ end [25, 43]. With the identification of *T. cruzi VIPER* SDRs, it is possible to observe that the *SIRE* element corresponds to the region encompassing A2 and B2 repeats (Fig. 2). This finding can open discussions about the *SIRE* origin (central or 5′ degeneration) and mobility capacity (mobile elements with the help of machinery provided by autonomous *VIPER* copies or just remnants of *VIPER* insertions).

In some *VIPER* copies, we observed the expected domains for YR (DNA_BRE_C super family) and RT/RH (RT_like super family and RNase_H_like super family) proteins. For the Gag-like protein, it was not expected to find a conserved domain. In this study, we found several different predicted domains (Additional file 3): 1) the SMC_N super family in copies from *A. deanei*, *C. fasciculata*, *C. mellificae,* and *T. cruzi* CL Brener Non-Esmeraldo-like; 2) the PHA03247 super family, downstream of the SMC_N domain in *C. mellificae*; and 3) the CwlO1 super family or AcrA in almost all *T. cruzi Dm*28c copies. Most of these domains are classified as a model that may span more than one domain and seems to be found in a vast number of nonrelated proteins [as checked in the Conserved Domain Database (CDD)]. Moreover, all these domains were predicted with high e-values (although below the threshold), indicating very low sequence similarity. Thus, it is difficult to determine whether the domains are indeed present in the proteins and the function they could perform (see Additional file 4 for more information).

In *T. cruzi Dm*28c, *T. theileri* and *Lep. pyrrhocoris* copies, we also found a CH-box (CX_2_CX_4_HX_4_C) motif that is often present in Gag proteins from retroviruses and LTR-retrotransposons [44, 45] and in some YR-retrotransposons [18]. This region is known to form a zinc finger with nucleic-acid-binding properties that is involved in packaging the RNA genome [46]. This motif was previously found in the Gag-like protein for the *T. cruzi* CL Brener *VIPER* consensus obtained by Lorenzi et al. (2006). In contrast, we were not able to find the major homology region (MHR - a 22-aa sequence located in the capsid domain of several Gag proteins) that was also predicted by these authors. We found no conserved CH-box in the copies from *B. saltans*, *A. deanei*, *C. fasciculata,* and *C. mellificae*.

### *TATE*, another YR retrotransposon in kinetoplastids

As shown in Fig. 1, *TATE* seems to have been completely lost in *Phytomonas sp*. isolates EM1 and Hart1, *Lo. passim*, *Lep. seymouri*, *L. tarentolae* and in most *Trypanosoma* species (except *T. theileri*). In several others, including most *Leishmania* species, *TATE* copies are possibly degenerate. We found potentially encoding copies of *TATE* in *B. saltans*, *C. fasciculata, C. mellificae, S. culicis, L. braziliensis* MHOM/BR/75/M2904*, Lep. pyrrhocoris* and *T. theileri.* Due to the phylogenetic proximity of *L. panamensis* and *L. peruviana* to *L. braziliensis* [40, 41], we expected to find similar patterns in the three species. However, most copies in *L. peruviana* and *L. panamensis* are in missing data regions. The same is found for the *L. braziliensis* MHOM/BR/75/M2903 strain. Table 2 and Additional file 5 summarize these results.

**Table 2:** Summary of *TATE* copy analyzes from the distinct genomes presenting at least one potentially encoding copy

Species	N^o^ of analyzed copies	N^o^ of copies with at least one potentially encoding ORF	N^o^ of copies with the three potentially encoding ORFs	N^o^ of potential encoding copies with YR and RT/RT related domains	N^o^ of copies with SDRs	Structure of SDRs size in base pairs, percentage of identity	N^o^ of putative autonomous copies*	Mean identity among copies in nu (total) and aa levels (for each ORF)**
*B. saltans*	5	2	2 *one ORF with the three genes	0	1	5′ → A1, A2 3′ → B1 → B2 A: 73 bp, 99%; B: 161 bp, 81%	0	nu: na Gag-like, YR, RT/RH (2, 1464 aa): 49%
*T. theileri*	15	7	4	0	1 (incomplete/missing data)	5′ → A1 3′ → A2 A: 91 bp, 100%;; B: missing data	0	nu: na Gag-like: na YR (3, 584 aa): 45.3% RT/RH (4, 698 aa): 86.9%
*C. fasciculata*	15	7	4	0	0	na	0	nu: na Gag -like (3, 240 aa): 31.1% YR (7, 727 aa): 44.8% RT/RH (3, 473 aa): 53.6%
*C. mellificae*	11	3	1	1	1 (incomplete/missing data)	5′ → A1 3′ → A2 A: 357 bp, 100%; B: missing data	0	nu: na Gag-like (2, 240 aa): 25.6% YR (3, 434 aa): 36.3% RT/RH: 1 sequence
*S. culicis*	15	1	1	0	0	na	0	1 sequence
*Lep. pyrrhocoris*	15	11	4	0	0	na	0	nu: na Gag-like (5, 194 aa): 29.7% YR (10, 468 aa): 48.9% RT/RH (8, 882 aa): 55.6%
*L. braziliensis*	15	15	7	6	1	5′ → A1 3′ → B1 → A2 → B2 A: 381 bp, 96%; B: 294 bp, 99%	?	nu: 90% Gag-like (8, 450 aa): 73.3% YR (15, 982 aa): 95.6% RT/RH (14, 954 aa): 96%

*putative autonomous copies are those containing the tree expected ORFs plus direct repeats

**identity was estimated only for regions with confident alignment. The number of sequences and the size of alignment is shown in parenthesis

? the copy where the SDRs were identified presents a frameshift mutation in the third ORF, however, we do not rule out the possibility that the genome could have additional copies that were not detected due to assembling issues

Two potentially encoding copies from *B. saltans* presented a single ORF structure, while copies from other species exhibited the expected three ORFs. The divergence of copies from the same species is high, except for *L. braziliensis*, which showed a mean of 90% nucleotide identity*.*

The *TATE* element seems to have a pattern of terminal repeats similar to that found for *VIPER*, although their identification was compromised in most species due to the proximity of copies to the end of contigs and due to the presence of more than one copy *in tandem* in the same scaffold. In *L. braziliensis*, at least one copy presented a pattern of SDRs, although the 3′ repeats were not shown in the Repbase *TATE* consensus sequence (Fig. 2). Moreover, we noticed more complex patterns of repetitions (Additional file 5) in some copies of *B. saltans* and *L. braziliensis*, but they could be a result of wrong genome assembly or chimeric natural sequences due to insertion of copies inside existent ones.

In *L. braziliensis,* we observed the expected domains for YR (DNA_BRE_C super family), and in the N-terminus region of several copies, this domain was preceded by a SPOR super family domain (predicted with low significance). This domain is involved in binding peptidoglycan and is found in proteins involved in sporulation and cell division, such as FtsN, DedD, and CwlM. Functional studies may help to address the role (if any) of this region. For ORF3, in addition to the RT_like superfamily, a Peptidase_A17 domain was found. Although this last domain is described as a homolog of aspartic proteinases at PFAM, it is, in fact, an RH domain and was inaccurately named [47]. The Gag-like protein of all *TATE* copies has no predicted domain and no clear CH-box.

### Evolution of *VIPER* and *TATE* in the trypanosomatid genomes

**Fig. 3:**
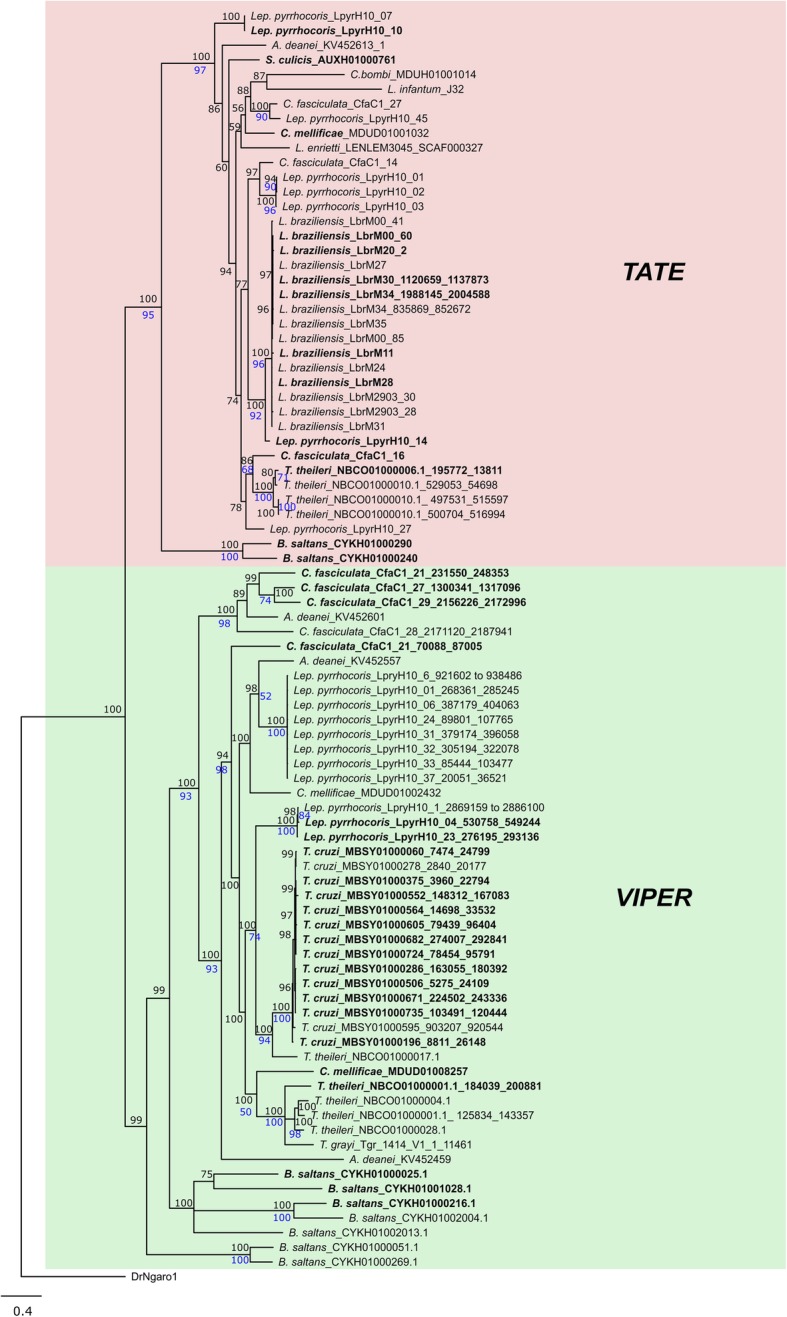
*VIPER* and *TATE* phylogenetic relationships among kinetoplastids. BA tree (mixed model + G, 336 aa) of *VIPER* and *TATE* copies of kinetoplastid species based on the RT/RH amino acid sequence. The RT/RH sequence of *DrNgaro1* was used as an outgroup. Posterior probability values obtained in the BA are indicated above the nodes, and clades with support less than 50% were collapsed; bootstrap values obtained in the ML analysis are indicated below the nodes in blue. Sequences that are part of putative encoding copies (with the three ORFs potentially encoding) are highlighted in bold

We performed phylogenetic reconstructions of *VIPER* and *TATE* elements based on the RT/RH and YR proteins. In both cases, both TEs revealed reciprocally monophyletic, although the YR tree presented a low phylogenetic resolution and few strongly established relationships (data not shown). Figure 3 shows the RT/RH majority-rule consensus tree obtained through BA, presenting the expected separation into two large clades corresponding to the different elements.

To understand the evolutionary history of these elements, we compared their phylogeny with the host species phylogenetic relationships, a standard procedure for TE evolutionary analysis [48–50]. For both TEs, *B. saltans* encompass the earlier offshoot, which is expected once this species is a nonparasitic close relative [51]. However, it is possible to find several incongruities in derived clades, such as the unexpected clustering of copies from distant species (for example, one *VIPER* copy of *C. mellificae* grouped with *T. theileri* and *T. grayi*) and scattered branching of copies of the same species (such as *TATE* copies from *Lep. pyrrhocoris* and *C. fasciculata* and *VIPER* copies from *Lep. pyrrhocoris, T. theileri, C. fasciculata, C. mellificae* and *A. deanei*). Those incongruities, however, cannot be explained by horizontal transfer, since the divergence among copies is not lower than expected (results not shown). This tree also shows cases of intragenomic mobilization and diversification (for example, *TATE* from *L. braziliensis* and *VIPER* from *T. cruzi* and *Lep. Pyrrhocoris*). Potentially encoding copies are present in several clades.

### Relationship of *VIPER* and *TATE* with other retroelements and YR-proteins

We found few significant hits for *VIPER* and *TATE* outside Kinetoplastida in the NCBI blastp searches. Hits for RT/RH and YR were recovered from *Oceanobacter sp* and *Candidatus Handelsmanbacteria*. We initially included these sequences in the phylogenetic analysis. However, the distribution of those revealed interspersed in the *TATE* and *VIPER* clades, raising the hypothesis of genomic sequence contamination that was strongly suggested after more in-depth investigations. Thus, these sequences were not used in the final phylogenies. Another significant hit for *VIPER* YR encompassed the sequence WP_106215579.1 from the bacterium *Kineococcus rhizosphaerae,* annotated as an integrase. We included this and other homologous proteins (retrieved after further blastp searches) in the phylogeny. Additional file 6 outlines the discussion concerning this issue.

**Fig. 4:**
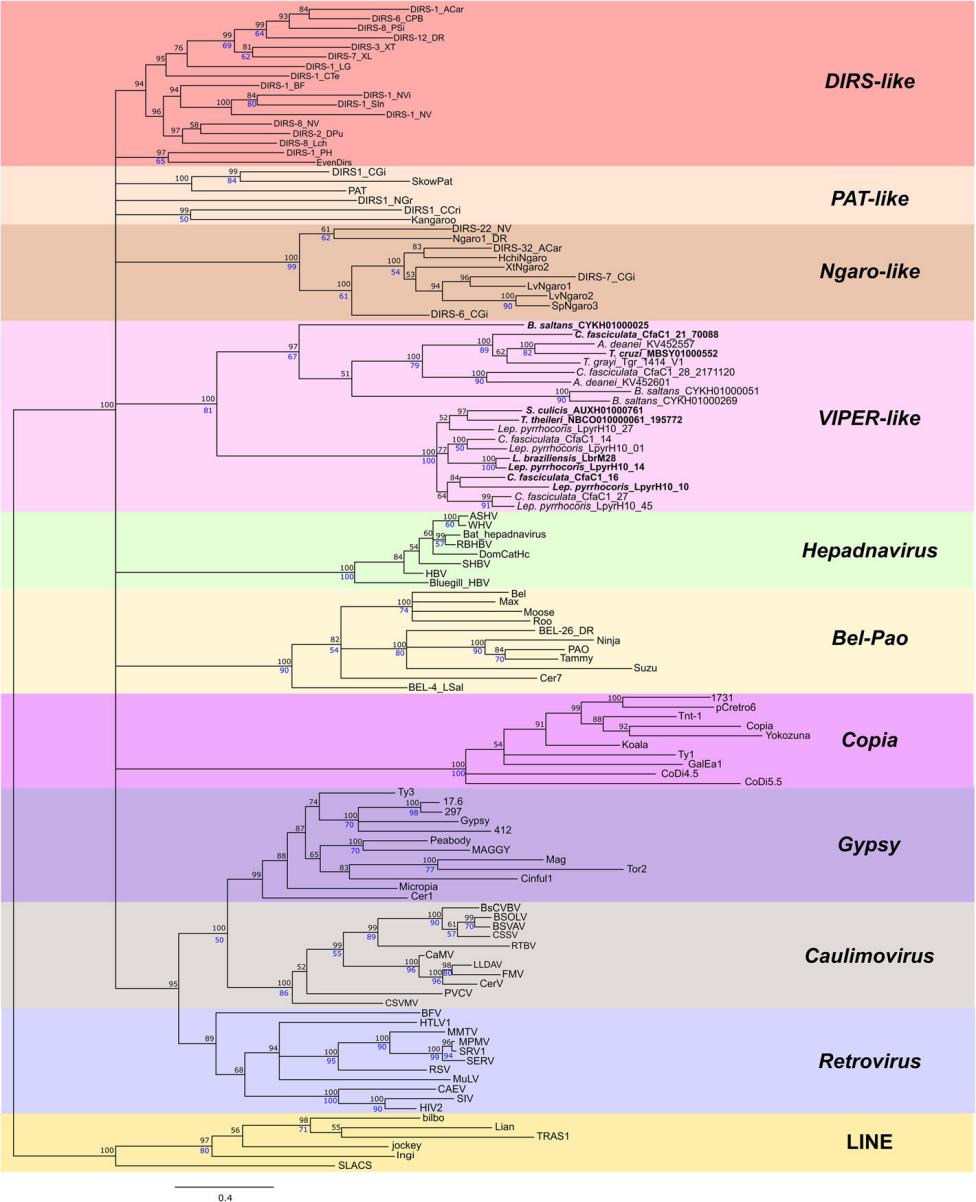
RT phylogenetic relationships. BA tree (mixed model + G, 197 aa) based on the RT amino acid sequences of different groups of retroelements. Possible encoding copies of *VIPER* and *TATE* are highlighted in bold. Posterior probability values obtained in the are indicated above the nodes, and clades with support less than 50% were collapsed; bootstrap values from ML analysis are presented in blue below the nodes (only values above 50). The tree was rooted with the LINE clade

**Fig. 5:**
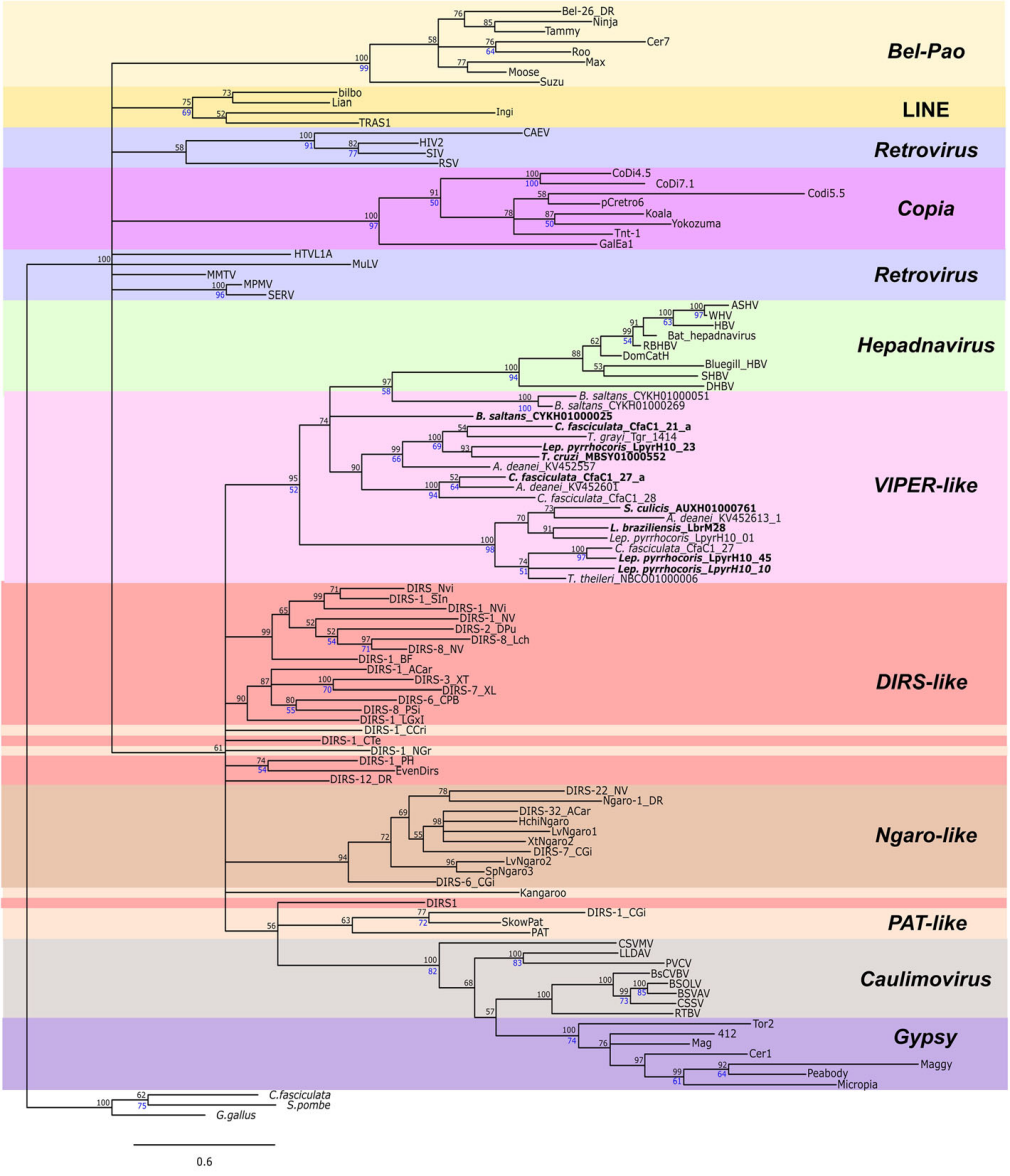
RH phylogenetic relationships. BA tree (mixed model + G, 146 aa) based on the RH amino acid sequences of different groups of retroelements. Possible encoding copies of *VIPER* and *TATE* are highlighted in bold. Posterior probability values are indicated above the nodes, and clades with support less than 50% were collapsed; bootstrap values from ML analysis are presented in blue below the nodes (only values above 50). The tree was rooted with the Eukaryote RH clade

**Fig. 6:**
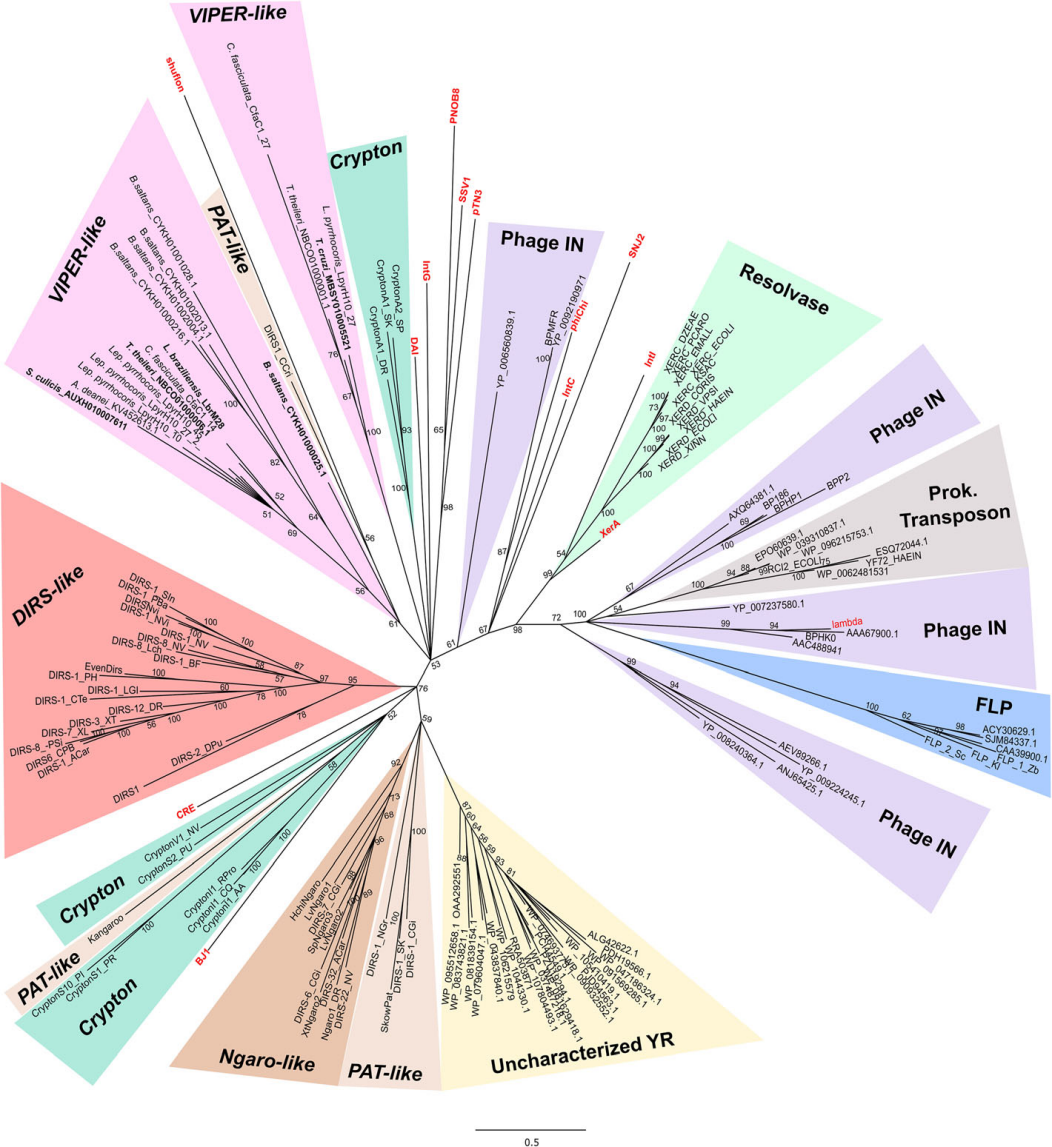
Phylogenetic relationships of YR family proteins. Unrooted BA tree (mixed model + G, 157 aa) based on the YR amino acid sequences from DIRS retrotransposons (*DIRS-like*, *PAT-like*, *Ngaro-like*, and *VIPER-like*) along with other YR family proteins: resolvase [site-specific recombinases involved in chromosome dimer resolution in archaea (XerA) and bacteria (XerC/D)], FLP (yeast Flp-like flippases), phage integrase, prokaryote transposons, Crypton, uncharacterized YR (proteins retrieve by blastp searches). YR sequences from the distinct groups shown by Wang et al. (2018) are in red: shufflon (shufflon-specific DNA recombinases); DAI (dusA-associated integrases); phage integrases; IntC (integrases from integrative and conjugative elements); IntI (integron integrases); IntG (genomic island integrases); XerA (site-specific recombinases involved in chromosome dimer resolution in archaea), Cre (phage P1-like recombinases); archaeal SSV-type, pNOB8-type and pTN3-type integrases; and integrases encoded by haloarchaeal SNJ2, BJ1-like and phiCh1-like viruses. Possible encoding copies of *VIPER* and *TATE* are highlighted in bold. Posterior probability values are indicated above the nodes, and clades with support less than 50% were collapsed; bootstrap values from ML analysis are presented in blue below the nodes (only values above 50)

ML and BA trees recovered similar topologies, whose results are presented in the form of the Bayesian majority rule consensus tree recovered for each major domain (Figs. 4, 5 and 6). Each of these trees summarizes the support values recovered through both ML and BA as distinct node labels.

Figure 4 shows the RT BA tree with a general topology less resolved than that found in other studies employing the same domain [26, 52], recovering only the well-supported clustering of *Gypsy*, *Caulimovirus,* and retroviruses. The lack of clustering of all YR elements could indicate an independent origin of these elements or merely the ancient separation of clades. As concerns the groups of YR elements, it was not possible to recover the monophyly either of *DIRS-like* (EvenDirs and DIRS-1_PH grouped in separate clades) or of *PAT-like* (three lineages), as also evidenced by Lorenzi et al. (2006) and Poulter and Butler (2015). In fact, the monophyly of these groups was only supported by Goodwin and Poulter (2004, 2005), who recovered a clade containing *Kangaroo* and *Pat* together with *DIRS-like* sequences in an RT/RH tree. Even so, *Ngaro-like* and *VIPER-like* sequences formed monophyletic groups (support of 100% BA, 99% ML and 100% BA, 81% ML, respectively). This finding confirms the close relationship of *VIPER* and *TATE* elements, both of which are encountered in trypanosomatids and share a similar structure despite sequence divergence.

The RH tree (Fig. 5) shows the positioning of retroviruses out of the Gypsy/caulimovirus clade [also seem by Malik and Eickbush (2001)]. Moreover, we observed an unexpected close relationship between *VIPER* and hepadnavirus, which are enveloped viruses with reverse-transcribed DNA genomes [53]. This possible relationship was never reported previously. In the RH tree from Malik and Eickbush (2001), *VIPER* sequences were not included, and in the Goodwin and Poulter (2004) RT/RH tree, both *VIPER* and Hepadnavirus sequences are located as polytomic branches together with *Ngaro-like*, caulimovirus/*Gypsy*, and *DIRS-like/PAT-like* sequences. It is possible that this relationship was masked in the last study due to the concatenation of RH with the RT domain. As occurred with RT, RH also shows unsatisfactory resolution to the clustering of elements belonging to different groups of YR elements, since *DIRS-like* and *PAT-like* sequences are scattered in the tree. Several of these results may be attributable to an outcome of the lower conservation of RH domains with the consequent loss of phylogenetic signal. Nevertheless, as the grouping of *VIPER* and hepadnavirus is strongly supported and was recovered under both ML and BA approaches, it is not possible to reject the possibility of changes/rearrangements in domains. Corroborating this similarity, when the RH portion of *Hepatitis B virus* (HBV) is isolated and used as a query on NCBI PSI-blastp searches [(excluding Hepadnaviridae (taxid:10404)], *B. saltans VIPER* sequences are retrieved in the third iteration.

For YR, in addition to *VIPER* and *TATE* sequences and the putative integrases mentioned above, we also included several sequences from different known groups of YR proteins. The phylogeny recovered was presented as unrooted, given the low resolution of the clades and the absence of a well-supported outgroup choice (Fig. 6). The relation of the *Crypton* group of DNA transposons with DIRS retroelements suggested by this tree had already been described [18], although we were not able to recover the monophyly of the different groups. This failure also occurred for *VIPER* and *TATE* sequences that were not recovered as reciprocally monophyletic groups. Interestingly, we can observe unpredicted grouping of YR elements with shufflon-specific DNA recombinase and some integrases, such as CRE (phage P1-like recombinases) and BJ1 (integrase from BJ1-like virus). Moreover, the YR tree suggests that those putative integrase sequences retrieved from NCBI after blastp searches likely correspond to some uncharacterized groups of YR proteins, since to the best of our knowledge, we have included representative sequences from the current characterized groups, and they did not cluster with any of them. In fact, these sequences constituted a single clade that seems to be closely related to some *PAT*-*like* and *Ngaro-like* elements.

## Discussion

### Influence of genome assemblies and TE detection methodology in the genomic analysis of *VIPER* and *TATE*

In this work, we used more than 40 Kinetoplastida genomes with different levels of assembly. Repetitive DNA sequences represent a challenge to the process of genome assembly, and information about these sequences is frequently incomplete. This problem can especially occur with next-generation sequencing approaches due to short reads [54, 55]. For *Lep. pyrrhocoris*, for example, despite the good quality of the assemblage at the chromosomal level, with 37X coverage [56], several TE copies were found near missing data regions. Another shortcoming of this kind of study is related to the incongruence between results performed with the same genome in different steps of data cleaning and assembly or with the same strain sequenced with distinct technologies. For example, the complete absence of *VIPER* evidenced in *T. rangeli* in this study contrasts with the remnants identified by other authors [38], which probably evaluated raw data. Moreover, in this study, we identified putative complete *VIPER* copies in the *T. cruzi Dm*28c strain that was sequenced using PacBio technology (GenBank assembly accession: GCA_002219105.2), which generates long reads and, consequently, better-quality assembly of the repetitive genome fraction [57]. When we previously analyzed the first available assembly for this strain (GenBank assembly accession: GCA_000496795.1 - sequenced by 454 technology), we were able to find encoding copies of *VIPER* but none with terminal repeat structure (data not shown). This finding suggests that we may be losing information about complete and putative encoding copies for other species. Nevertheless, despite these limitations, genomic data continue to be a valuable source for TE studies, being widely used to better understand the elements in the genomic contexts of different organisms [58–62], and in this report, we describe significant findings of *VIPER* and *TATE* elements.

Several programs and pipelines are now available to detect and annotate repetitive sequences, mainly using detection based on similarity, structure or repetitiveness [63]. These tools are crucial for large-scale approaches and for providing an overall view of the TE composition of genomes. Here, we study two TE families applying a less automated search based on similarity, though with a careful evaluation of copies nevertheless. The most challenging work was the definition of the boundaries of the elements. Thus, we also used a pipeline for the automatic identification of TEs in the two most relevant genomes (*T. cruzi* for *VIPER* and *L. braziliensis* for *TATE*) to confirm our manual evaluations. We did not observe advantages of using these pipelines to identify the boundaries of the elements, since even in these cases, we had to proceed with manual inspection of identified copies, and in most cases, they were predicted to be shorter than those we found.

### *VIPER* and *TATE*: ancient residents and survivors of kinetoplastids genomes

We presented the first investigation of *VIPER* and *TATE* elements from a wide-scale phylogenetic perspective. Our in silico searches show a discontinuous distribution of both TEs in the host species, whose evolution was better understood with the phylogenetic analyses. In this study, we adopted the most parsimonious scenario according to the current data for all evolutionary inferences, implying that the future addition of new genome sequences or new species may lead to changes in assumptions.

Initially, Lorenzi et al. (2006) proposed that *VIPER* would have colonized the genomes of *Trypanosoma* species after the separation of this genus and *Leishmania* due to the absence of *VIPER* in the *Leishmania* species that were previously analyzed. Our data fully challenge this scenario because we found potentially encoding copies or remnants of *VIPER* not only in *B. saltans* [as already reported by Jackson et al. (2016)] but also in species of the Leishmaniinae subfamily. Although the presence of these elements in *B. saltans* could be indicative of horizontal transfer, sequence divergences support vertical transmission. In fact, this pattern of evolution seems to be shared for several gene families where *B. saltans* appears to have retained many genes that were present in its ancestor with the trypanosomatids and that ended up being lost discontinuously in some specific groups throughout the evolution of trypanosomatids [64, 65].

Due to the divergence found among *VIPER* and *TATE* copies within the same species and the pattern of separation of copies into distinct clades, we can suggest the existence of numerous cases of ancestral polymorphism. However, the topology of the tree indicates that most of the *VIPER* and *TATE* polymorphisms arose after the separation of trypanosomatids with the ancestor of *B. saltans*. Despite the inconclusions in some points of the phylogeny, we can suggest several independent loss events of the elements along with the trypanosomatid evolution. The absence of significant *hits* supports the complete degeneration of *TATE* in most *Trypanosoma* species and *VIPER* in most *Leishmania* species, which can be taken with some confidence once several genomes have an adequate assembly level.

In this study, for the first time, we described potentially complete and encoding copies of *VIPER* in the *T. cruzi Dm*28c strain, including terminal repeats. Only degenerate copies were found in the *T. cruzi* CL Brener genome in this and in other works [25–27]. We cannot conclude whether the differences between *T. cruzi* strains are due to genome assembly problems or due to genetic differences between strains. In the course of writing, another *T. cruzi Dm*28c PacBio assembly was published by Berná et al. (2018) [66]. These researchers found approximately 200 copies of *VIPER* that were considered defective. In a brief search of the potentially encoding *VIPER* identified in our work against this genome, we were able to find matches with 100% identity, indicating that the assignment of *T. cruzi Dm*28c *VIPER* copies as defective by these authors was probably due to differences in interpretation. Additionally, these authors did not identify the terminal repeat regions, and the copies were considered to have 3.423 bp, considerably smaller than the complete copies that we found. Although the DNA_BRE_C domain was predicted only with less significant e-values (CD-search e-value of approximately 0.1), the composition and conservation of ORFs and the presence of putative SDRs suggest that these *T. cruzi Dm*28c *VIPER* copies are potentially active. RNA-Seq reads mapping *VIPER* were found for *T. cruzi Dm*28c during epimastigote growth in vitro [67]. However, we need to be cautious concluding TE activity in trypanosomatids based only on this type of data, since those reads could be only a transitory molecule resulting from global RNA expression. On the other hand, peptides from Gag-like protein were detected during the differentiation process from *T. cruzi Dm*28c noninfective epimastigotes into infective metacyclic trypomastigotes [68], a stronger sign of possible activity. In addition, we found potential encoding *VIPER* copies in some other species.

We also obtained significant results for the *TATE* element, such as the terminal repeat pattern in *L. braziliensis* and the presence of potentially encoding copies in some other species. The *L. braziliensis* genome presents good coverage and assembly (Peacock et al. 2007), but the repetitive nature of *TATE* and its disposition *in tandem* made the analysis more difficult. The complete copy that we found in this genome contains a frameshift at the end of the third ORF; however, we believe that additional full and potentially active copies could be found in an improved genome assembly. Assembling issues may also have hindered better characterization in other species. Recent analysis of differential expression across the life cycle stages of *L. braziliensis* reveals that *TATE* RNAs are overrepresented in amastigote and metacyclic forms [69]. *TATE* sequences also constituted 38% of small interfering RNAs (siRNAs) associated with *L. braziliensis* Argonaute1 protein [70]. The presence of siRNA from *TATE* in this species suggests that an endogenous RNAi pathway could be responsible for controlling the transposition of this element [70, 71].

Considering the putative complete *VIPER* and *TATE* copies, we encountered some terminal repeats sharing 100% identity. For *Ngaro-like* and *PAT-like* elements, a model of reverse transcription was proposed including steps of annealing between portions of the repeats [23, 24]. This model implies that repeat sequences A1 and A2 and B1 and B2 should be at least nearly identical in the time of insertion. If *VIPER* and *TATE* possess a similar reverse transcription process, the conservation of repeats is also an indication of recent activity.

In conclusion, the presence of *VIPER* and *TATE* in *B. saltans* suggests that the two elements were already present in the last common ancestor of the studied species more than 400 mya and have remained active in some species. This “survival” for such an extended period raises questions about the coevolution of these sequences with their host genomes, such as the way that these sequences have escaped from inactivation and how the genomes control these sequences. Alternatively, these elements could have experienced reactivation events. Furthermore, with so many millions of years of coevolution, it is natural to think about the impact of these elements on the evolution of species and how TEs may have contributed to the organization and functionality of the kinetoplastid genomes.

### Putative modular evolution of YR retroelements

Analysis of RT, RH and YR domains was performed separately, which is important because we already had suggestions of incongruent relationships among the proteins [26] and because it is known that retrotransposons and retroviruses have a modular evolution profile with recurrent events of domain rearrangements or even a reticulated origin of its components [72–74]. The ability to recover the evolutionary history of these sequences depends on the time in which these elements arose and the selective pressures to which they were exposed. These details have a direct influence on the quality and reliability of sequence alignments. According to our previous assumption, we observed no resolution for some clades, possibly due to the lack of phylogenetic signal that may reflect very ancient separation events, possibly before the divergence of the major eukaryotic groups. Thus, it is quite likely that most of the incongruent results found in this study in the comparisons between topologies obtained for different domains are an outcome of a weak phylogenetic signal. This effect seems to be the case in, for example, the recurrent polytomy detected for *DIRS*-*like* and *PAT*-*like* and by the absence of monophyly of *VIPER*-*like* sequences in the RH and YR phylogenies. Despite this finding, we showed here for the first time that *VIPER* and *TATE* elements form a monophyletic group in the RT phylogeny, suggesting a common origin of both TEs. This result questions the recent findings of Pita et al. (2019), which suggested *TATE* as a distinct group from other DIRS elements based on an RT tree [10]. Nevertheless, it is not clear if authors have used *VIPER* sequences in their phylogeny.

Although the monophyly of *VIPER-like* could be confidently assigned by the most conserved of the evaluated domains (RT), the origin of these domains and their relationship with other retrotransposons were not clearly established. In fact, considering the three evaluated domains, there are no significant results supporting either independent or common origins for different YR retroelements. In this sense, YR-element groups were all placed at the same level in a highly polytomous RT phylogeny. In the RH phylogeny, YR-elements grouped with *Gypsy-caulimovirus* and *Hepadnavirus*, and in the YR phylogeny, they grouped with *Crypton*, the uncharacterized YRs and some other integrase/recombinase sequences. In the last case, *Crypton* revealed polyphyletic, which was already expected, since previous phylogenetic evaluations recovered four groups with weakly supported relationships [75]. Poulter and Butler (2015) suggested that the origin of YR retrotransposons possibly occurred many times, resulting in three groups: *DIRS*-*like*, *PAT*-*like* and *Ngaro*. The independent origin hypothesis was also proposed by Goodwin and Poulter (2004) and Lorenzi et al. (2006). In this study, we were not able to find strong evidence of independent origins for the different YR elements; therefore, our results cannot rule out a scenario of common ancient origin.

The contrasting clustering of YR elements among RT, RH and YR suggests that they could share a modular evolutionary pattern with distinct sources for the different domains. In this sense, whereas the RT-domain may share common origins with LTR-retrotransposons *Gypsy* (as evidenced more clearly by other authors [18, 26]), the RH-domain evidenced a close relationship with *Hepadnavirus*. Moreover, the YR domain of YR elements may stem from *Crypton* or some integrase/recombinase sequences (or even may have given rise to them). Such a modular evolution pattern was already proposed for YR retroelements by Poulter and Butler (2015), which suggested that these elements had an origin from a combination of *Crypton* DNA elements and an RT/RH from a *Gypsy* LTR-retrotransposon. Although our data do not disagree with this proposal in its total, we can suggest alternative scenarios, such as the origin of their YR directly from some prokaryotic or viral integrases/recombinases. Similarly, the reverse relation, where viral and prokaryotic YR sequences originate from a YR element, could also have occurred. More in-depth studies of homologous sequences to YR retroelements and other retrotransposons will possibly help to refine this scenario.

Another intriguing relationship recovered by this study is that between the RH domain of *VIPER-like* TEs and hepadnaviruses. In fact, the phylogeny revealed that *VIPER-like* RH sequences are more closely related to hepadnavirus than to the other YR retroelements. This finding may suggest that RH sequences suffered additional rearrangements along the evolutionary history of YR retroelements. According to Lauber et al. (2017), hepadnavirus and nackedvirus (a nonenveloped HBV-related family of fish viruses [76]) separated more than 400 mya before the rise of tetrapods. This evolutionary period coincides with the minimum time of origin of *VIPER* and *TATE* elements, suggesting a possible shared source of the RH domain of *VIPER-like* and the ancestor of hepadnavirus and nackednavirus.

In general, as we did not find clear evidence of independent origins of *VIPER-like* from the other YR retroelements, we support the maintenance of the DIRS group of retrotransposons. Based on the scenario presented in this study and others, we propose dividing the YR-containing retroelements into four groups, which were named according to the first described element (these names were also the ones chosen by other authors) followed by the indication “like”: *DIRS-like*, *PAT -like*, *Ngaro-like* and *VIPER-like*. This proposition complements other works that mention the classification of these elements [18, 29, 77]. *PAT-like, Ngaro-like,* and *VIPER-like* elements show a similar terminal repeat pattern, whereas *DIRS-like* elements have important repeat structure differences [18] and consequently differ in the mechanism of reverse transcription [22, 23]. Since other studies revealed that *PAT-like* and *DIRS-like* are phylogenetically closely related [17, 20, 24], their separation is mainly due to the structural difference of the terminal repeats [18]. According to the classification of Wicker et al. (2007), the subgroups correspond to superfamilies from the order DIRS. Although the order DIRS is not demonstrated to be monophyletic, this classification helps to refer to YR-containing retrotransposons.

## Conclusions

This report presents a comprehensive description of the structure, distribution, and evolution of *VIPER* and *TATE* retrotransposons in trypanosomatid species, suggesting that these elements are notably ancient components of those genomes and have survived and remained active in some species or were reactivated during host species evolution. We also investigated the relationship of these elements with other retrotransposons. Our data do not support independent origins for different YR retroelements but suggest the occurrence of ancient exchanges of domains. We also proposed to separate the elements of the order DIRS into four subgroups according to phylogenetic findings and sequence structure: *DIRS-like, PAT-like, Ngaro-like,* and *VIPER-like.*

## Methods

### Genomes and in silico searches

The genomes were retrieved from TriTrypDB [78], or NCBI [79], and their information is available in Additional file 7. Local tblastn searches were performed against each genome using the amino acid sequences of the three proteins of *VIPER* and *TATE*, whose canonical sequences were obtained from Repbase database [80]. Hits were considered significant when their respective e-values were lower than e^− 10^. To confirm the identity of sequences, we used CENSOR software [81], a tool that screens query sequences against the Repbase database.

### Characterization of copies

All putative *VIPER* and *TATE* copies were retrieved together with 8 kb of flanking regions (whenever available) using an *in-house* Perl script. Redundancy was further eliminated using the CD-HIT-est tool [82] and through manual review. Up to fifteen of the most conserved copies of each species were carefully analyzed by individual manual inspection. The analyzed copies of *VIPER* and *TATE* are provided in Additional files 8 and 9, respectively. The presence of ORFs was analyzed using the NCBI ORFfinder tool. NCBI CD-search [83] was used to check for domains in the predicted protein sequences. RT/RH and YR ORFs were identified mainly by their similarity to *VIPER* and *TATE* canonical proteins or by the predicted domains. ORF 1 or *gag-like* was predicted primarily by its proximity or overlap with ORF 2 and by the lack of orthologues in other species. Copies were considered potentially encoding when they presented three ORFs encoding proteins with significant size (> 300 aa for gag-like, > 250 aa for YR and > 600 aa for RT/RH). Putative complete copies of *VIPER *and *TATE* possessing repeats are provided in Additional files 10 and 11, respectively.

*VIPER* and *TATE* DNA sequences were aligned with MUSCLE program [84], and the alignments were visually inspected to identify the limits of the retrotransposon copies. To verify the presence of terminal repeat structures, the potentially complete copies were analyzed by blastn with the parameters “align two or more sequences” and “somewhat similar sequences (blastn)” using the same sequence as query and subject.

To evaluate our analysis strategy, mainly avoiding erroneous determination of element limits, we also used an automated pipeline to search for *VIPER* in *T. cruzi Dm*28c and *TATE* in *L. braziliensis* MHOM/BR/75/M2904. We used the detection TE programs (based on assembled genomes) from PiRATE, as automated in Galaxy platform [63, 85].

To detect *VIPER* activity in *T. cruzi Dm*28c, we analyzed the presence of RNA-seq reads and peptides from *VIPER* in results of published transcriptomic [67] and proteomic studies of this strain [68]. As most *VIPER* copies are annotated as hypothetical proteins, we retrieved all TriTrypDB gene IDs and UniProt IDs from *VIPER* copies and searched for them in the tables containing all genes and proteins identified in the studies using *in-house* Python code.

### Phylogenetic analyses of *TATE* and *VIPER*

To understand the evolutionary history of *VIPER* and *TATE*, we inferred the phylogeny of RT/RH and YR amino acid sequences separately. All ORFs presenting significant sizes were included, and the sequence DrNgaro (AAN71721.1) from *Danio rerio* was used as an outgroup. Amino-acid sequences were aligned with the MUSCLE program, as available in MEGA 7 [96]. For RT/RH, only the regions encompassing domains 1–7 [52] and domains D10 to D134 [74], respectively, were trimmed from the original sequences, whereas for YR the region encompassing boxes 1 and 2 [26], added to the intervening sequence was evaluated. The alignments are available in the Additional files 12 and 13.

Phylogenetic analyses were then performed for each of these datasets using Bayesian Analysis (BA) in MrBayes 3.2.6 [86] under a mixed amino acid model. The Markov Chain Monte Carlo (MCMC) of the BA was run for 10,000,000 generations, sampling trees every 1000 generations, and burning 25% of the initial results. Additionally, a maximum likelihood (ML) analysis was performed under the rapid bootstrap algorithm, which also searches for best-scoring ML trees. This bootstrap analysis was conducted for 1000 replicates using RAxML-HPC2 implemented on CIPRES [87] following the amino acid substitution model with the lowest Bayesian Information Criterion (BIC), as measured in ModelTest-NG (Darriba et al. 2019) (LG + G4 + F and WAG+G4 for RT/RH and YR, respectively). Phylogenetic trees resulting from BA and ML searches were visualized and edited in FigTree 1.4.2 [88] and Evolview tool, respectively.

### Evolution of DIRS elements

Looking for *VIPER-* and *TATE*-related sequences, online blastp searches were performed on NCBI against the nonredundant protein sequences (nr) database using the canonical sequences of *VIPER* and *TATE* RT/RH and YR proteins as queries. Sequences with significant e-values (e < 10^− 5^) were included in the phylogenies. Additionally, for YR, other related sequences were retrieved by blastP searches and included in the phylogeny. Full information about these searches is available in Additional file 6.

To contemplate the diversity of YR-containing TEs in the phylogenies, we analyzed all TEs assigned as autonomous DIRS in the Repbase (note that it includes all YR-containing elements). A total of 413 sequences were retrieved and analyzed for the presence of ORFs and domains. Of those sequences, 128 presented the three expected conserved domains (RT-, RH-, and YR-related domains) and were selected. We then filtered this dataset, eliminating monophyletic sequences from the same species with less than 35% divergence. This preliminary analysis was performed using Neighbor-Joining under the Poisson correction method, as performed in MEGA 7 [89]. Approximately 30 sequences remained in the dataset after this initial filtering.

The RT and RH datasets were further complemented by the inclusion of representative sequences from each of the following related groups: LINE, *Gypsy, Copia, Bel-Pao*, hepadnavirus, caulimovirus, and retrovirus (see Additional file 14). For RH, it was also possible to include eukarya sequences. For YR, we added the sequences found in kinetoplastids, the related proteins found by blastp searches and several known related proteins representatives of distinct groups {Flippase, Phage integrase, Resolvase (XerC and XerD) and Prokaryotic Transposase [also used by Lorenzi et al. (2006)]}. We also included one representative sequence of each of the major clades recovered by Wang et al. (2018) [90] (see Additional file 15).

For the RT-related and RNA-directed RNA polymerase sequences, the region encompassing domains 1–7 [52] was first trimmed from the original sequences and then subjected to two multiple progressive alignments approaches: 1) the first performed with ClustalW under the BLOSUM protein weight matrix, individually for each major sampling group (*DIRS/Pat/Ngaro*, *VIPER/TATE*, LINE, *Gypsy, Copia, Bel-Pao*, hepadnavirus, caulimovirus, retrovirus); 2) the second performed with MAFFT [91] for the entire set of sequences, employing the E-INS-i iterative refinement method with the help of DASH (Database of Aligned Structural Homologs) homologous structures. In both steps, alignment was checked regarding homology assignments within each of the seven RT domains, and variable regions between domains were withdrawn, whereas gaps were filled with “X”.

For RH, two alignment approaches were also performed using ClustalW and Muscle after an initial trimming of the region encompassing domains D10 to D134 [74]. For YR, boxes 1 and 2 [26] were first located, and the surrounding N-terminus and C-terminus regions of both domains were deleted. The alignment was then performed with the same criteria described above using ClustalW and Muscle but with a distinct grouping strategy at the first step [DIRS/Pat/Ngaro, VIPER/TATE, flippase, phage integrase, resolvase (XerC and XerD) and prokaryotic transposase].

Phylogenetic analyses were performed under BA and ML, as described above. Whereas mixed models were employed for BA analyses, ML analyses were performed under individual amino acid substitution models selected in ModelTest-NG (LG + G4, RTREV+G4 + F and LG + G4, for RH, RT and YR, respectively). RT analyses were rooted using the non-LTR LINE clade (*ingi, Jockey, bilbo, Lian, TRAS1* and *SLACS*) as an outgroup, as previously supported [52], whereas RH phylogenies were rooted by eukarya sequences, following Malik and Eickbush (2001). For YR, the phylogenies were presented as unrooted phylograms. All the alignments are provided in Additional files 16, 17 and 18.

## Additional files

Additional file 1: Table. The number of significant hits (e-value cut-off of 10^− 10^) found on tblastn searches for *VIPER* and *TATE* proteins against each genome. (PDF 20 kb)

Additional file 2: Table. Summary of the analyzes of the 15 most conserved copies of *VIPER* and *TATE* elements in each species. (XLSX 98 kb)

Additional file 3: Table. Summary of main features of potentially encoding *VIPER* copies. (PDF 72 kb)

Additional file 4: Additional information. Domains predicted for *VIPER* and *TATE* proteins (PDF 87 kb)

Additional file 5: Table. Summary of main features of potentially encoding *TATE* copies (PDF 71 kb)

Additional file 6: Additional information. Evaluation of *VIPER* and *TATE* similar sequences outside Kinetoplastida. (PDF 147 kb)

Additional file 7: Table. List of genomes used for in silico searches. (PDF 25 kb)

Additional file 8: Sequences. *VIPER* copies analyzed in all species are provided together with the flanking region (TXT 2890 kb)

Additional file 9: Sequences. *TATE* copies analyzed in all species are provided together with the flanking region (TXT 2700 kb)

Additional file 10: Sequences. Putative complete *VIPER* copies (TXT 52 kb)

Additional file 11: Sequences. Putative complete *TATE* copies (TXT 31 kb)

Additional file 12: Alignments. Alignment of RT/RH sequences of *VIPER* and TATE (FAS 30 kb)

Additional file 13: Alignments. Alignment of YR sequences of *VIPER* and *TATE* (FAS 15 kb)

Additional file 14: Table. List of sequences used for RT phylogeny (PDF 29 kb)

Additional file 15: Table. List of sequences used for YR phylogeny (PDF 29 kb)

Additional file 16: Alignments. Alignment of RT sequences of different groups of retroelements (FAS 24 kb)

Additional file 17: Alignments . Alignment of RH sequences of different groups of retroelements (FAS 17 kb)

Additional file 18: Alignments . Alignment of YR sequences of DIRS retrotransposons (*DIRS-like, PAT-like, Ngaro-like,* and *VIPER-like*) along with other YR family proteins (FAS 23 kb)

## Data Availability

Genomic coordinates of *VIPER* and *TATE* copies analyzed here are available in the Additional file 2, and the sequences are available in Additional files 8 and 9.
