# Supplementary material for: Study of VIPER and TATE in kinetoplastids and the evolution of tyrosine recombinase retrotransposons
Source: Mob DNA. 2019 Aug 5;10:34. doi: 10.1186/s13100-019-0175-2 (PMC6681497; doi:10.1186/s13100-019-0175-2)
Supplement: Supplementary file 1 — Table. The number of significant hits (e-value cut-off of 10− 10) found on tblastn searches for VIPER and TATE proteins against each genome. (PDF 20 kb) [file 13100_2019_175_MOESM1_ESM.pdf]

The number of significant hits (e-value cut-off of 10^{-10}) found on tblastn searches for *VIPER* and *TATE* proteins against each genome.

	VIPER			***TATE***		
	Gag	YR	RT/RH	Gag	YR	RT/RH
Bodo saltans Lake Konstanz	0	0	10	0	5	0
Angomonas deanei ATCC PRA-265	0	3	3	0	5	12
Blechomonas ayalai B08-376	0	0	1	0	36	77
Crithidia. fasciculata strain Cf-Cl	0	25	41	0	151	107
C. bombi IL132	0	2	12	0	19	36
C. mellificae ATCC 30862	0	13	33	0	8	9
Endotrypanum monterogeii strain LV88	0	0	0	12	45	25
Leishmania aethiopica L147	0	0	0	0	0	6
L. amazonensis MHOM/BR/71973/M2269	0	0	0	0	3	1
L. arabica strain LEM1108	0	0	0	0	3	1
L. braziliensis MHOM/BR/75/M2903	0	0	7	31	77	89
L. braziliensis MHOM/BR/75/M2904	0	0	11	34	50	60
L. donovani BPK282A1	0	0	0	0	2	4
L. enriettii strain LEM3045	0	0	0	0	45	67
L. gerbilli strain LEM452	0	0	0	0	6	4
L. infantum JPCM5	0	0	0	0	4	4
L. major strain Friedlin	0	0	0	0	1	3
L. mexicana MHOM/GT/2001/U1103	0	0	0	0	3	4
L. panamensis MHOM/COL/81/L13	0	0	6	12	17	21
L. peruviana PAB-4377_V1	0	0	10	27	94	109
L. tarentolae Parrot-TarII	0	0	0	0	0	0
L. tropica L590	0	0	0	0	6	4
L. turanica strain LEM423	0	0	0	0	3	0
Leishmania sp. MAR LEM2494	0	0	1	0	18	17
Leptomonas pyrrhocoris H10	0	29	69	2	53	43
Lep. seymouri ATCC 30220	0	0	0	0	0	0
Lotmaria passim ATCC PRA-422	0	0	0	0	0	0
Phytomonas francai	0	0	0	0	3	7
Phytomonas sp. isolate EM1	0	0	0	0	0	0
Phytomonas sp. isolate Hart1	0	0	0	0	0	0
Strigomonas culicis TCC012E	0	0	1	0	10	31
Trypanosoma brucei brucei TREU927	0	36	58	0	0	0
T. brucei gambiense DAL972	0	17	24	0	0	0
T. brucei Lister strain 427	0	26	30	0	0	0
T. congolense IL3000	0	44	80	0	0	0
T. cruzi Dm28c PB1	752	617	1425	0	0	0
T. cruzi CL Brener Esmeraldo	272	254	578	0	0	0
T. cruzi CL Brener Non-Esmeraldo	321	264	663	0	0	0
T. cruzi marinkellei strain B7	369	398	671	0	0	0
T. equiperdum OVI V2	0	15	25	0	0	0
T. evansi strain STIB 805	0	17	28	0	0	0
T. grayi ANR4	0	72	159	0	0	0
T. rangeli SC58	0	0	0	0	0	0
T. theileri Edinburgh	7	39	76	0	17	19
T. vivax Y486	0	101	256	0	0	0
