# Supplementary material for: Study of VIPER and TATE in kinetoplastids and the evolution of tyrosine recombinase retrotransposons
Source: Mob DNA. 2019 Aug 5;10:34. doi: 10.1186/s13100-019-0175-2 (PMC6681497; doi:10.1186/s13100-019-0175-2)
Supplement: Supplementary file 3 — Table. Summary of main features of potentially encoding VIPER copies. (PDF 72 kb) [file 13100_2019_175_MOESM3_ESM.pdf]

Summary of main features of potentially encoding *VIPER* copies

***Scaffold/contig*_position**	**ORF1<br>Position<br>Domains**	**ORF2<br>Position<br>Domains**	**ORF3<br>Position<br>Domains**	**Repeats<br>Basic structure size, identity**
		B. saltans		
CYKH01000025.1_4008-9037	1-1335<br>N	1136-2419<br>DNA_BRE_C	1615-4173<br>RT_*like*<br>RNase	5’ – absent<br>3’ →A1→B1→A2→B2<br>A: 285 bp, 96%;<br>B: 198 bp, 97%
CYKH01002013.1_423-4623	1-1206<br>N	1281-2189<br>DNA_BRE_C	1031-3778<br>RNase	5’ →A1→A2<br>3’ – missing data<br>A: 83 bp, 93%
CYKH01001028.1_613-5045	1-1203<br>N	1079-2095<br>DNA_BRE_C	1288-3819<br>N	5’ – absent<br>3’ – absent
CYKH01000216.1_959-6047	1-1236<br>N	1600-2472<br>DNA_BRE_C	1503-4130<br>RNase	5’ →A1<br>3’ →B1→B2→A2<br>A: 83 bp, 100%;<br>B: 57 bp, 100%
		T. cruzi*		
MBSY01000735.1_110090-114144	1-1341<br>CwlO1	1342-2359<br>DNA_BRE_C	1218-2163<br>RT_*like*<br>RNase	5’ →A1<br>3’ →B1→A2→B2<br>A: 173 bp, 100%;<br>B: 219 bp, 100%
		T. theileri		
NBCO01000001.1_ 190527-200565	1-1266<br>N	1399-2359<br>N	1577-3551<br>RT-*like*	5’ – absent<br>3’ →A1→A2<br>A: 95 bp, 100%
NBCO01000017.1_ 258857-262458	1-906<br>N	1079-2095<br>DNA_BRE_C	2021-3601<br>RT_*like*<br>RNase	5’ – absent<br>3’absent
		A. deanei		
KV452601.1_66-3952	1-1281<br>SMC_N	1308-2220<br>N	1898-3821<br>RVT_1<br>RNase	5’ – missing data<br>3’ – missing data
		C. fasciculata		
CfaC1_21_76128-86740	1-1182<br>SMC_N	2057-2888<br>DNA_BRE_C	1120-4573<br>RT-*like*<br>RNase	5’ →A1<br>3’ →B1→A2→B2<br>A: 136 bp, 99%;<br>B: 288 bp, 100%
CfaC1_27_1306980-1317400	1-1251<br>N	1356-2184<br>N	1247-3782<br>Herpes_ICP4<br>RT-*like*<br>RNase	5’ →A1<br>3’ →B1→A2→B2<br>A: 144 bp, 100%;<br>B: 308 bp, 100%
CfaC1_29_2163302-2173637	1-1227<br>N	939-1860<br>N	1238-3641<br>RT-*like*<br>RNase	5’ – absent<br>3’ – absent
CfaC1_21_237095-247550	1-1416<br>GRS1	2387-3302<br>N	1917-4911<br>RT-*like*<br>RNase	5’ →A1<br>3’ →B1→A2→B2<br>A: 414 bp, 93 %;<br>B: 294 bp, 99%
		Lep. pyrrhocoris		
LpyrH10_23_286594-293282	1-1191<br>N	1187-2447<br>N	1105-4090<br>RT-*like*<br>RNase	5’ – absent<br>3’ – absent
LpyrH10_04_536700-547229	1-1689<br>N	1685-2945<br>N	1603-4588<br>RVT_1<br>RNase	5’ – absent<br>3’ – absent
		C. mellificae		
MDUD01008257.1_1-4861	1-1695<br>SMC_N<br>Atrophin_1	1442-2966<br>N	3112-4621<br>RT-*like*<br>RNase	5’ –missing data<br>3’ – absent

Note: The position in the scaffold/contig refers to the position of the copy in the scaffold (first nucleotide of ORF 1 to the last nucleotide of ORF3). The position of the ORFs refers to their position within the copy, starting with the first position of the ORF1. Different repetitions are represented by different letters (A and B). The positions (5' or 3' end) of the repeat fragments are indicated and the fragments are represented in order. The size and identity of repeats are indicated. The presence of protein domains is indicated; N - no predicted domain;

* Only one copy of *T. cruzi* was represented since there is high conservation among copies.
