# Supplementary material for: Study of VIPER and TATE in kinetoplastids and the evolution of tyrosine recombinase retrotransposons
Source: Mob DNA. 2019 Aug 5;10:34. doi: 10.1186/s13100-019-0175-2 (PMC6681497; doi:10.1186/s13100-019-0175-2)
Supplement: Supplementary file 4 — Additional information. Domains predicted for VIPER and TATE proteins (PDF 87 kb) [file 13100_2019_175_MOESM4_ESM.pdf]

Domains predicted for VIPER and TATE proteins

We were able to find the expected protein domains for YR and RT/RH proteins from *VIPER* and *TATE*, whose function is already known. One significant issue concerning YR-containing retrotransposons is the function of the ORF1 product called Gag-like. We know that Gag protein is required for the formation of Virus-Like Particles (VLPs) in retroviruses and LTR-retrotransposons [1], which makes us wonder whether Gag-like protein found in *VIPER*, *TATE* and other DIRS elements would also form VLPs. Besides positioning of ORF, there is no significant amino acid similarity of *VIPER*, and *TATE* Gag-like with retroviral Gag and no specific protein domain was expected to be found based in previous studies. Although not expected, several distinct domains were found nearly in the same region of *VIPER* Gag-like. CwlO1 super family domain, an uncharacterized N-terminal domain of peptidoglycan hydrolase CwlO. Some Ngaro-like elements present a hydrolase ORF encoding the SGNH_hydrolase domain [2]. However, we do not see any clear relation since CwlO1 super family domain is found several other types of proteins [as seem in Conserved Domain Database (CDD)], like as glucan-binding protein B, carbohydrate porin, IS66 family transposable element, ABC transporter, among others. Additionally, AcrA domain (Multidrug efflux pump subunit AcrA - membrane-fusion protein) is also predicted for some *T. cruzi* copies in the same region as the CwlO1 super family domain was predicted for other copies (note that copies are less than 4% divergence). SMC_N super family domain, found at the N-terminus of SMC proteins (structural maintenance of chromosomes) and PHA03247 (large tegument protein UL36; Provisional) is classified as a model that may span more than one domain and seems to be found in a vast number of non-related proteins. Moreover, all these domains were predicted with high significant e-value, although below the threshold, indicating very low sequence similarity. Thus, it is hard to say that they are indeed present

in the proteins and the function that they could perform. The same can be suggested for the prediction of SPOR super family domain in the *TATE* YR. This domain is involved in binding peptidoglycan and is found in proteins involved in sporulation and cell division such as FtsN, DedD, and CwlM. Functional studies of these proteins may help to accurate address their role.

References:

1. Roth JF. The yeast Ty virus-like particles. Yeast [Internet]. 2000;16:785–95. Available from: http://www.ncbi.nlm.nih.gov/pubmed/10861903

2. Poulter RTM, Butler MI. Tyrosine Recombinase Retrotransposons and Transposons. Microbiol Spectr [Internet]. 2015;3:MDNA3-0036-2014. Available from: http://www.ncbi.nlm.nih.gov/pubmed/26104693
