# Supplementary material for: Study of VIPER and TATE in kinetoplastids and the evolution of tyrosine recombinase retrotransposons
Source: Mob DNA. 2019 Aug 5;10:34. doi: 10.1186/s13100-019-0175-2 (PMC6681497; doi:10.1186/s13100-019-0175-2)
Supplement: Supplementary file 5 — Table. Summary of main features of potentially encoding TATE copies (PDF 71 kb) [file 13100_2019_175_MOESM5_ESM.pdf]

Summary of main features of potentially encoding *TATE* copies

*Scaffold/contig*_position	ORF1<br>Position<br>Domain	ORF2<br>Position<br>Domain	ORF3<br>Position<br>Domain	Repeats<br>Basic structure<br>size, identity
		B. saltans		
CYKH01000290.1_1157-6382		1-5226<br>DNA_BRE_C<br>RVT_1		5’ →A1→A2<br>3’ →B1→B2<br>A: 73 bp, 99%; B: 161 bp, 81%
CYKH01000240.1_371-4765		1-4395<br>DNA_BRE_C<br>RVT_1		5’ – absent<br>3’ – absent
		C. fasciculata		
CfaC1_16_593910-598902	1-1225<br>N	1269-3471<br>DNA_BRE_C	2960-4994<br>RT_like<br>DNA_pol3	5’ – absent<br>3’ – absent
		C. mellificae		
MDUD01001032.1_377-5156	1-987<br><br>N	1338-3360<br><br>DNA_BRE_C	3389-5156<br>RT_like<br>RNase	5’ →A1<br>3’ →A2<br>A: 357 bp, 100%<br>B: missing data
		S. culicis		
AUXH01000761.1_1187-6266	1-840<br>N	933-3987<br>Atrophin-1	3566-6266<br>RT_like	5’ →A1<br>3’ →A2<br>A: 346 bp, 99%
		Lep. pyrrhocoris		
LpyrH10_03_995010-1005122	1-1095<br>N	674-3938<br>DNA_BRE_C	3151-5869<br>N	5’ – absent<br>3’ – absent
LpyrH10_45_4163-13642	1-1200<br>N	1623-3486<br>DNA_BRE_C	2732-5318<br>RT_like<br>dnaA	5’ – absent<br>3’ – absent
LpyrH10_10_418649-428385	1-1014<br>N	969-3291<br>DNA_BRE_C	3317-5138<br>RT_like	5’ – absent<br>3’ – absent
		L. braziliensis		
LbrM.11_536345-553559	1-1218<br>N	1311-4259<br>DNA_BRE_C	Interrupted<br>ORF	5’ →A1<br>3’ →B1→A2→B2<br>A: 381 bp, 96%<br>B: 294 bp, 99%
LbrM.30_1128659-1142875	1-1218<br>N	1311-4245<br>DNA_BRE_C	3356-6218<br>RT_like<br>RNase	5’ A1→B1→A2<br>3’ →C→B2<br>A: 375 bp, 98%<br>B: 637 bp, 94%<br>C: 293 bp, 98%
LbrM.20.2_676198-690033	1-1218<br>N	1311-4260<br>DNA_BRE_C<br>SPOR	3371-5837<br>RT_like<br>RNase<br>GcrA	5’ ←A1<br>3’ ←A2<br>A: 71 bp, 100%
LbrM.34_19966225-2010719	1-3642<br>DNA_BRE_C<br>SPOR		2754-5616<br>RT_like<br>RNase	5’ →A1<br>3’ →A2<br>A: 382 bp, 89%
		T. theileri		
NBCO01000006.1_199922-210051	1-1104<br>N	1281-3762<br>DNA_BRE_C	3014-5981<br>RT_like	5’ →A1<br>3’ →A2<br>A: 91 bp, 100%

Note: The position in the scaffold/contig refers to the position of the copy in the scaffold (first nucleotide of ORF 1 to the last nucleotide of ORF3). The position of the ORFs refers to their position within the copy, starting with the first position of the ORF1. Different repetitions are represented by different letters (A, B, C). The positions (5' or 3' end) of the repeat fragments are indicated and the fragments are represented in order. The size and identity of repeats are indicated. The presence of protein domains is indicated; N - no predicted domain;
