# Supplementary material for: Study of VIPER and TATE in kinetoplastids and the evolution of tyrosine recombinase retrotransposons
Source: Mob DNA. 2019 Aug 5;10:34. doi: 10.1186/s13100-019-0175-2 (PMC6681497; doi:10.1186/s13100-019-0175-2)
Supplement: Supplementary file 6 — Additional information. Evaluation of VIPER and TATE similar sequences outside Kinetoplastida. (PDF 147 kb) [file 13100_2019_175_MOESM6_ESM.pdf]

Evaluation of *VIPER* and *TATE* similar sequences outside Kinetoplastida.

Using blasp searches against the nr database of NCBI, we found some significant hits that suggested similarities between *VIPER* and *TATE* Reverse Transcriptase/RNase H (RT/RH) and Tyrosine Recombinase (YR) and some proteins from *Oceabacter sp*. (RT - PHR97723.1, PHR97648.1, PHR97720.1, PHR97545.1; YR - PHR97649.1, PHR97720.1, PHR97545.1, PHR97880.1, PHR94983.1) and *Candidatus Handelsmanbacteria* (RT - OGG55579.1; YR - OGG55578.1). The reciprocal blast searches show Kinetoplastid sequences from the second hit.

Although unexpected, this similarity was quite interesting, since it could represent the outcome of horizontal transfer. Thus, the sequences were included in the RT phylogeny. Nevertheless, the resultant tree evidenced a scattered distribution of these sequences inside the *VIPER-like* clade (Figure 1, below).

Trying to understand if the sequences are in fact part of *Oceabacter sp*. and *Candidatus Handelsmanbacteria* bacterium genomes, we retrieved their genome assemblies from NCBI:

1) *Oceanobacter sp*. (g-proteobacteria); GCA_002733645.1; submitter: University of Southern California; date: 2017/10/26; metagenome-source: marine metagenome.
2) *Candidatus Handelsmanbacteria* bacterium; GCA_001781055.1; submitter: Banfield Lab, University of California, Berkeley; date: 2016/10/19; metagenome-source: subsurface metagenome.

We observed in the tblastn results that the sequences similar to *VIPER* and *TATE* from these species are located mainly in the extremities of short contigs (Tables 1 and 2, below), so it is not possible to support that these sequences are actually part of these genomes. Moreover, additional searches performed with other species closely related to *Oceanobacter sp*. (*Oceanobacter kriegii* DSM 6294, GCA_000422845.1; *Oleibacter marinus,* GCA_900156675.1; *Thalassolituus oleivorans* MIL-1, GCA_000355675.1) and with a different genome assembly of *Candidatus Handelsmanbacteria* bacterium (GCA_003562965.1) did not result in significant hits. The sequences, however, are clearly DIRS retrotransposons since they present RT and YR domains.

Although we do not discard other explanations, taking all the considerations into account, we have not strong evidence that *Oceanobacter sp* and *Candidatus Handelsmanbacteria* possess sequences similar to *VIPER* and *TATE*. It is possible that

the results represent contamination on genome sequence databases. The distribution of sequences in the phylogeny could suggest the sequences are from a kinetoplastida species that was not sequenced yet, probably a free-living species which may have been sampled in the metagenomes in question.

Another unexpected significant hit encompassed sequence WP_106215579.1 that shows similarity to *VIPER* YR and is annotated as an integrase, from a bacterium, *Kineococcus rhizosphaerae*. We found homologous sequences of this gene in other two *Kineococcus* species, *Kineococcus xinjiangensis,* and *Kineococcus radiotolerans*.

Trying to understand these sequences, we retrieved their genome assemblies from NCBI:

1) *Kineococcus radiotolerans* SRS30216; GCA_000017305.1; submitter: US DOE Joint Genome Institute; date: 2008/08/14.
2) *Kineococcus xinjiangensis;* GCA_002934625.1; submitter: DOE Joi*nt Genome Institute; date: 2018/02/21.*
3) *Kineococcus rhizosphaerae*; GCA_003002055.1; submitter: DOE Joint Genome Institute; date: 2018/03/15.

In this case, there is no strong evidence for sequence contamination, and we believe these sequences are, in fact, part of the genomes. Nevertheless, we did not find any evidence that these sequences are *DIRS* retrotransposons or DNA transposons. They are not repetitive, and they have no repeats like TIRs or TSDs. Trying to understand the relationship of these sequences with *VIPER-like* elements, we performed a BlastP search of WP_106215579.1 protein, and we retrieve the first 250 sequences (e-value range from 0.0 - 1e-55). These sequences were aligned in Muscle[1], and a neighbor-joining tree was obtained on MEGA 7 [2]. Only one sequence from each well-supported clade was maintained, totaling 23 sequences that were used in the final YR tree showed in the main text.

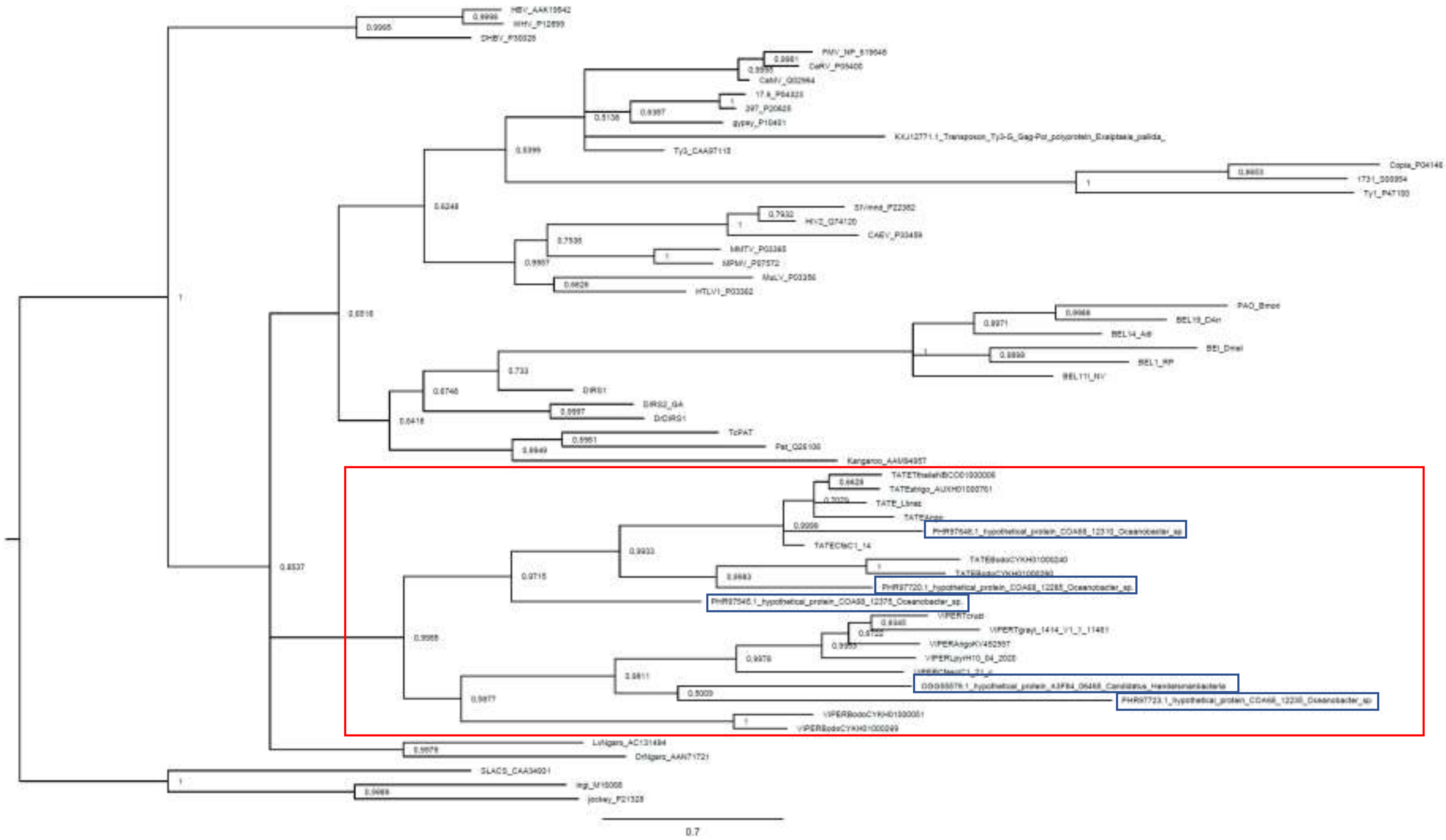

Figure 1: Phylogenetic relationships recovered through Bayesian analysis of retroelements RT domain showing the sequences from *Oceanobacter sp* (blue boxes) scattered into the *VIPER-like* clade (red box). Posterior probabilities are shown near nodes.

Table 1: Summary of tblastn results of *VIPER* and *TATE* Reverse transcriptase (RT) and Tyrosine recombinase (YR) coding sequences in *Oceanobacter sp*. genome.

Protein /Element	Contig/Scaffold	Size Contig/ Scaffold	Identity	Align length	Start query	End query	Start subject	End subject	e-value
VIPER RT	NVWL01000035.1_cds_PHR97723.1_314	1665	25.85	468	483	909	1662	304	2,00E-24
VIPER RT	NVWL01000085.1_cds_PHR94983.1_1960	4032	24.55	440	504	910	1452	196	6,00E-14
VIPER RT	NVWL01000036.1_cds_PHR97720.1_324	5646	25.00	388	559	909	2639	1545	5,00E-11
VIPER RT	NVWL01000041.1_cds_PHR97545.1_543	4026	28.57	217	503	710	1528	926	1,00E-09
VIPER RT	NVWL01000032.1_cds_PHR97879.1_3428	1578	24.00	375	481	836	10714	9644	2,00E-09
TATE RT	NVWL01000037.1_cds_PHR97648.1_491	1671	35.64	811	163	941	4754	2502	6,00E-111
TATE RT	NVWL01000036.1_cds_PHR97720.1_324	5646	26.83	615	343	940	3065	1464	5,00E-28
TATE RT	NVWL01000032.1_cds_PHR97879.1_3428	1578	28.64	426	339	752	10903	9734	3,00E-18
TATE RT	NVWL01000085.1_cds_PHR94983.1_1960	4032	24.91	542	399	914	1548	190	2,00E-10
TATE RT	NVWL01000041.1_cds_PHR97545.1_543	4026	26.62	417	391	770	1633	497	4,00E-10
TATE YR	NVWL01000037.1_cds_PHR97649.1_492	2517	37.81	320	659	973	5320	4385	6,00E-47
TATE YR	NVWL01000036.1_cds_PHR97720.1_324	5646	31.83	311	657	960	4166	3279	3,00E-28
TATE YR	NVWL01000032.1_cds_PHR97880.1_3429	1479	30.05	203	764	964	11583	11011	8,00E-12
TATE YR	NVWL01000041.1_cds_PHR97545.1_543	4026	28.62	283	683	960	3001	2192	1,00E-11
TATE YR	NVWL01000085.1_cds_PHR94983.1_1960	4032	31.63	215	763	966	2583	1999	3,00E-11

Table 2: Summary of tblastn results of *VIPER* Reverse transcriptase (RT) and Tyrosine recombinase (YR) coding sequences in the *Candidatus Handelsmanbacteria bacterium* genome.

Protein /Element	Contig/Scaffold	Size Contig/ Scaffold	Identity	Align length	Start query	End query	Start subject	End subject	e-value
VIPER YR	MFKF01000070.1	5402	28.87	284	63	318	2426	3268	8e-018
VIPER RT	MFKF01000070.1	5402	29.81	416	519	911	3952	5166	2e-042

References

1. Edgar RC. MUSCLE: multiple sequence alignment with high accuracy and high throughput. Nucleic Acids Res. 2004;32:1792–7.

2. Kumar S, Stecher G, Tamura K. MEGA7: Molecular Evolutionary Genetics Analysis Version 7.0 for Bigger Datasets. Mol Biol Evol. 2016;33:1870–4.
