# Supplementary material for: Study of VIPER and TATE in kinetoplastids and the evolution of tyrosine recombinase retrotransposons
Source: Mob DNA. 2019 Aug 5;10:34. doi: 10.1186/s13100-019-0175-2 (PMC6681497; doi:10.1186/s13100-019-0175-2)
Supplement: Supplementary file 7 — Table. List of genomes used for in silico searches. (PDF 25 kb) [file 13100_2019_175_MOESM7_ESM.pdf]

List of genomes used for *in silico* searches.

Source: TritrypDB			
**Organism**	**Release/Version**	**Summary**	**Contact/Data Source**
B. ayalai B08-376	Version: 2017-01-18	*Blechomonas ayalai* B08-376 Sequence and annotation	Vyacheslav Yurchenko
C. fasciculata strain Cf-Cl	Version: 2015-06-01	*Crithidia fasciculata* Cf-C1 sequence and annotation	Stephen M. Beverley
E. monterogeii strain LV88	Version: 2015-01-16	*Endotrypanum monterogeii* LV88 Genome sequence and annotation	Stephen M. Beverley
L. aethiopica L147	Version: 2014-12-16	Genome sequence and annotation of *Leishmania aethiopica* L147	Stephen M. Beverley
*L. amazonensis*MHOM/BR/71973/M2269	Version: 2013-07-25	Genome sequence of *Leishmania amazonensis* MHOM/BR/71973/ M2269	Diana Bahia
L. arabica strain LEM1108	Version: 2014-12-16	Genome sequence and annotation for *Leishmania arabica* strain LEM1108	Stephen M. Beverley
L. braziliensis MHOM/BR/75/M2903	Version: 2014-12-16	*Leishmania braziliensis* M2903 Genome sequence and annotation	Stephen M. Beverley
L. braziliensis MHOM/BR/75/M2904	Version: 2016-05-28	*Leishmania braziliensis* M2904 (MHOM/BR/75M2904) sequence and annotation from GeneDB.	Pathogen Sequencing Unit
L. donovani BPK282A1	Version: 2016-05-28	*Leishmania donovani* BPK282A1 sequence and annotation	Matthew Berriman
L. enriettii strain LEM3045	Version: 2014-12-16	*Leishmania enriettii* strain LEM3045 sequence and annotation	Stephen M. Beverley
L. gerbilli strain LEM452	Version: 2014-12-16	Genome sequence and annotation for *Leishmania gerbilli* strain LEM452	Stephen M. Beverley
L. infantum JPCM5	Version: 2015-12-07	Genome sequence and annotation for *Leishmania infantum* JPCM5 (MCAN/ES/98/LLM-877)	Pathogen Sequencing Unit
L. major strain Friedlin	Version: 2016-05-28	Genome sequence and annotation for *Leishmania major* Friedlin.	Pathogen Sequencing Unit
L. mexicana MHOM/GT/2001/U1103	Version: 2016-05-28	Genome sequence and annotation for *Leishmania mexicana* U1103	Pathogen Sequencing Unit
L. panamensis MHOM/COL/81/L13	Version: 2014-12-16	*Leishmania panamensis* L13 Genome sequence and annotation	Stephen M. Beverley
L. pyrrhocoris H10	Version: 2015-05-05	*Leptomonas pyrrhocoris* H10 sequence and annotation	Julius Lukes
L. seymouri ATCC 30220	Version: 2015-05-06	*Leptomonas seymouri* ATCC 30220 sequence and annotation	Vyacheslav Yurchenko
L. sp. MAR LEM2494	Version: 2014-12-16	*Leishmania sp.* MAR LEM2494 Genome sequence and annotation	Stephen M. Beverley
L. tarentolae Parrot-TarII	Version: 2011-06-22	*Leishmania tarentolae* Parrot-TarII sequence and annotation	Jacques Corbeil
L. tropica L590	Version: 2014-12-16	Genome sequence and annotation of *Leishmania tropica* L590	Stephen M. Beverley
L. turanica strain LEM423	Version: 2014-12-16	Genome sequence and annotation for *Leishmania turanica* strain LEM423	Stephen M. Beverley
T. brucei brucei TREU927	Version: 2016-05-19	*Trypanosoma brucei* TREU927 sequence and annotation	Pathogen Sequencing Unit
T. brucei gambiense DAL972	Version: 2016-05-28	Genome sequence and annotation for *Trypanosoma brucei* gambiense	Pathogen Sequencing Unit
T. brucei Lister strain 427	Version: 2010-10-20	Genome sequence and annotation for *Trypanosoma brucei* strain Lister 427	Pathogen Sequencing Unit
T. congolense IL3000	Version: 2016-06-06	*Trypanosoma congolense* IL3000 sequence and annotation	Matthew Berriman

T. cruzi CL Brener Esmeraldo-like	Version: 2015-12-07	Genome sequence and annotation for *Trypanosoma cruzi* CL Brener.	Pathogen Sequencing Unit
T. cruzi CL Brener Non-Esmeraldo-like	Version: 2015-12-07	Genome sequence and annotation for the non-Esmeraldo haplotype of *Trypanosoma cruzi* CL Brener	GeneDB
T. cruzi marinkellei strain B7	Version 1.0	*Trypanosoma cruzi* marinkellei sequence and annotation	Oscar Frazen
T. evansi strain STIB 805	Version: 2014-06-03	*T. evansi* STIB 805 sequence and annotation	Achim Schnaufer
T. grayi ANR4	Version: 2014-06-17	*Trypanosoma grayi* ANR4 sequence and annotation	Mark Field
T. rangeli SC58	Version: 2013-10-30	*Trypanosoma rangeli* SC58 sequence and annotation	Edmundo Carlos Grisard
T. vivax Y486	Version: 2016-06-06	Genome sequence and annotation for *Trypanosoma vivax* Y486	Pathogen Sequencing Unit
**Source: NCBI**			
**Organism**	**GenBank assembly accession**	**Genome coverage/Assembly level**	**Submitter**
Angomonas deanei ATCC PRA-265	GCA_001659865.1	344x/Scaffold	Heinrich Heine University Duesseldorf
Bodo saltans Lake Konstanz	GCA_001460835.1	170x/ Contig	Wellcome Trust Sanger Institute
Crithidia bombi IL132	GCA_002216585.1	100x/ Scaffold	University of Massachusetts
Crithidia mellificae ATCC 30862	GCA_002216565.1	100x/ Scaffold	University of Massachusetts
Leishmania peruviana PAB-4377_V1	GCA_001403675.1	59x/Chromosome	UFMG
Lotmaria passim ATCC PRA-422	GCA_002216525.1	100x/ Scaffold	University of Massachusetts
Phytomonas francai	GCA_001766655.1	200x/ Scaffold	University of Oxford
Phytomonas sp. isolate EM1	GCA_000582765.1	20x/ Scaffold	Genoscope CEA
Phytomonas sp. isolate Hart1	GCA_000982615.1	20x/ Scaffold	Genoscope CEA
Strigomonas culicis TCC012E	GCA_000482145.1	23x/ Contig	Virginia Commonwealth University
Trypanosoma cruzi Dm28c PB1	GCA_002219105.2	150x/ Contig	Instituto Carlos Chagas - ICC/Fiocruz-PR
Trypanosoma equiperdum OVI V2	GCA_001457755.2	104x/ Scaffold	ANSES
Trypanosoma theileri Edinburgh	GCA_002087225.1	100x/ Scaffold	University of Edinburgh
