# Supplementary material for: Study of VIPER and TATE in kinetoplastids and the evolution of tyrosine recombinase retrotransposons
Source: Mob DNA. 2019 Aug 5;10:34. doi: 10.1186/s13100-019-0175-2 (PMC6681497; doi:10.1186/s13100-019-0175-2)
Supplement: Supplementary file 14 — Table. List of sequences used for RT phylogeny (PDF 29 kb) [file 13100_2019_175_MOESM14_ESM.pdf]

List of sequences used for RT and RH phylogenies.

Name	Organism	ID NCBI/Uniprot	Classification
HBV	*Hepatitis B virus*	YP_009173866.1	*Hepadnavirus*
WHV	*Woodchuck hepatitis vírus 59*	P12899	*Hepadnavirus*
DomCatH	*Domestic cat hepadnavirus*	NC_040719	*Hepadnavirus*
Bluegill_HBV	*Bluegill hepatitis B virus*	NC_030445	*Hepadnavirus*
ASHV	*Arctic ground squirrel hepatitis B*	U29144	*Hepadnavirus*
RBVHB	*Roundleaf bat hepatitis B virus*	KC790376	*Hepadnavirus*
Bat_hepadnavirus	*Bat hepadnavirus*	MG600411	*Hepadnavirus*
SHBV	*Shrew hepatitis B virus*	MH484442	*Hepadnavirus*
DHBV	*Duck hepatitis B virus*	P30028.1	*Hepadnavirus*
LvNgaro1	*Lytechinus variegatus*	BK001253	*Ngaro-like*
HchiNgaro	*Hynobius chinensis*	GAQK01079872	*Ngaro-like*
XtNgaro2	*Xenopus tropicalis*	AC175582	*Ngaro-like*
DIRS-7_CGi	*Crassostrea gigas*	Repbase	*Ngaro-like*
DIRS-32_ACar	*Anolis carolinensis*	Repbase	*Ngaro-like*
DIRS-6_CGi	*Crassostrea gigas*	Repbase	*Ngaro-like*
SpNgaro3	*Strongylocentrotus purpuratus*	AAGJ04006304	*Ngaro-like*
DIRS-22_NV	*Nematostella vectensis*	Repbase	*Ngaro-like*
LvNgaro2	*Lytechinus variegatus*	AGCV01398517	*Ngaro-like*
Ngaro1_Dr	*Danio rerio*	Repbase	*Ngaro-like*
MuLV	*Murine leukemia virus*	P03356.3	*Retrovirus*
HTLV1	*Human T-cell lymphotrophic virus*	P03362.3	*Retrovirus*
MMTV	*Mouse mammary tumor virus*	P03365.3	*Retrovirus*
MPMV	*Mason-Pfizer monkey virus*	P07572.1	*Retrovirus*
SIV	*Simian immunodeficiency virus*	P22382.2	*Retrovirus*
HIV2	*Human immunodeficiency virus type*	Q74120.3	*Retrovirus*
CAEV	*Caprine arthritis encephalitis virus*	P33459.1	*Retrovirus*
BFV	*Bovine foamy virus*	NC_001831	*Retrovirus*
SERV	*Simian endogenous retrovirus*	U85505	*Retrovirus*
SRV1	*Simian retrovirus 1*	P04025	*Retrovirus*
RSV	*Rous sarcoma virus*	BAD98246	*Retrovirus*
FMV	*Figwort mosaic virus*	NP_619548.1	*Caulimovirus*
BSOLV	*Banana streak OL virus*	AJ002234	*Caulimovirus*
BsCVBV	*Bougainvillea spectabilis chlorotic*	EU034539	*Caulimovirus*
CSSV	*Cacao swollen shoot virus*	L14546	*Caulimovirus*
BSVAV	*Banana streak virus*	AY750155	*Caulimovirus*
CSVMV	*Cassava vein mosaic virus*	U59751	*Caulimovirus*
LLDAV	*Lamium leaf distortion associated*	NC_010737	*Caulimovirus*
PVCV	*Petunia vein clearing virus*	NC_001839	*Caulimovirus*
RTBV	*Rice tungro bacilliform virus*	X57924	*Caulimovirus*
CerV	*Carnation etched ring virus*	P05400.1	*Caulimovirus*
CaMV	*Cauliflower mosaic virus*	Q02964.1	*Caulimovirus*
17.6	*Drosophila melanogaster*	P04323.1	*Gypsy*
297	*Drosophila melanogaster*	P20825.1	*Gypsy*
Gypsy	*Drosophila melanogaster*	P10401.1	*Gypsy*
Ty3	*Saccharomyces cerevisiae*	CAA97115.1	*Gypsy*
MAGGY	*Pyricularia grisea*	L35053.1	*Gypsy*

Peabody	*Pisum sativum*	AF083074	*Gypsy*
412	*Drosophila melanogaster*	X04132	*Gypsy*
Micropia	*Drosophila melanogaster*	X14037	*Gypsy*
Cer1	*Caenorhabditis elegans*	U15406	*Gypsy*
Mag	*Bombyx mori*	X17219	*Gypsy*
Tor2	*Oikopleura dioica*	AY634223	*Gypsy*
Cinful1	*Zea mays*	AF049110.1	*Gypsy*
Copia	*Drosophila melanogaster*	P04146.3	*Copia*
1731	*Drosophila melanogaster*	S00954	*Copia*
Ty1	*Saccharomyces cerevisiae*	P47100.3	*Copia*
CoDi5.5	*Thalassiosira pseudonana*	EU432490	*Copia*
GalEa1	*Eumunida annulosa*	EU097705	*Copia*
Koala	*Oryza australiensis*	DQ365823	*Copia*
Tnt-1	*Nicotiana tabacum*	X13777	*Copia*
Yokozuna	*Bombyx mori*	AB014676	*Copia*
pCretro6	*Phanerochaete chrysosporium*	DQ097838	*Copia*
CoDi4.5	*Thalassiosira pseudonana*	EU432485	*Copia*
CoDi7.1	*Phaeodactylum tricornutum*	EU432499	*Copia*
Kangaroo	*Volvox carteri f. nagariensis*	AAM94957.1	*PAT-like*
PAT	*Panagrellus redivivus*	Q26106	*PAT-like*
DIRS-1_CCri	*Chondrus crispus*	Repbase	*PAT-like*
DIRS-1_CGi	*Crassostrea gigas*	Repbase	*PAT-like*
SkowPAT	*Saccoglossus kowalevskii*	ACQM01123180	*PAT-like*
DIRS-1_NGr	*Naegleria gruberi*	Repbase	*PAT-like*
DIRS-1_PH	*Parhyale hawaiensis*	Repbase	*DIRS*
EvenDIRS	*Echinogammarus veneris*	GARO01000003	*DIRS*
DIRS-1_ACar	*Anolis carolinensis*	Repbase	*DIRS*
DIRS-6_CPB	*Chrysemys picta bellii*	Repbase	*DIRS*
DIRS-8_PSi	*Pelodiscus sinensis*	Repbase	*DIRS*
DIRS-12_DR	*Danio rerio*	Repbase	*DIRS*
DIRS-3_XT	*Xenopus tropicalis*	Repbase	*DIRS*
DIRS-7_XL	*Xenopus laevis*	Repbase	*DIRS*
DIRS-1_LG	*Lottia gigantea*	Repbase	*DIRS*
DIRS-1_CTe	*Capitella teleta*	Repbase	*DIRS*
DIRS-1_BF	*Branchiostoma floridae*	Repbase	*DIRS*
DIRS-1_NV	*Nematostella vectensis*	Repbase	*DIRS*
DIRS-1_NVi	*Nasonia vitripennis*	Repbase	*DIRS*
DIRS-1_SIn	*Solenopsis invicta*	Repbase	*DIRS*
DIRS-2_DPu	*Daphnia pulex*	Repbase	*DIRS*
DIRS-8_NV	*Nematostella vectensis*	Repbase	*DIRS*
DIRS-8_Lch	*Latimeria chalumnae*	Repbase	*DIRS*
DIRS1	*Dictyostelium discoideum*	M11339	*DIRS*
DIRS_Nvi	*Nasonia vitripennis*	Repbase	*DIRS*
PAO	*Bombyx mori*	Repbase	*Bel-Pao*
BEL	*Drosophila melanogaster*	U23420.1	*Bel-Pao*
BEL-26_DR	*Danio rerio*	Repbase	*Bel-Pao*
Ninja	*Drosophila simulans*	D83207.1	*Bel-Pao*
Tammy	*Antheraea mylitta*	AF530470.1	*Bel-Pao*
Max	*Drosophila melanogaster*	AJ487856.1	*Bel-Pao*

Cer7	*Caenorhabditis elegans*	AF014939.1	*Bel-Pao*
Moose	*Anopheles gambiae*	AF060859.1	*Bel-Pao*
Roo	*Drosophila melanogaster*	AY180917.1	*Bel-Pao*
Suzu	*Takifugu rubripes*	AF537216.1	*Bel-Pao*
BEL-4_LSal	*Lepeophtheirus salmonis*	Repbase	*Bel-Pao*
Ingi	*Trypanosoma brucei*	M16068	*LINE*
Jockey	*Drosophila melanogaster*	P21328	*LINE*
Lian	*Aedes aegypti*	Repbase	*LINE*
Bilbo	*Drosophila subobscura*	Repbase	*LINE*
TRAS1	*Bombyx mori*	Repbase	*LINE*
SLACS	*Trypanosoma brucei*	CAA34931.1	*LINE*
S.pombe	*Schizosaccharomyces pombe*	NP_596126.1	*RH outgroup*
G.gallus	*Gallus gallus*	NP_990329.1	*RH outgroup*
C.fasciculata	*Crithidia fasciculata*	Q07762.1	*RH outgroup*
