# Supplementary material for: Study of VIPER and TATE in kinetoplastids and the evolution of tyrosine recombinase retrotransposons
Source: Mob DNA. 2019 Aug 5;10:34. doi: 10.1186/s13100-019-0175-2 (PMC6681497; doi:10.1186/s13100-019-0175-2)
Supplement: Supplementary file 15 — Table. List of sequences used for YR phylogeny (PDF 29 kb) [file 13100_2019_175_MOESM15_ESM.pdf]

List of sequences used for YR phylogeny.

Name	Organism	ID NCBI/Uniprot	Classification
FLP_Kl	*Kluyveromyces lactis*	P13783.1	*Flipases*
FLP_1_Zb	*Zygosaccharomyces bisporus*	P13784.1	*Flipases*
FLP_2_Sc	*Saccharomyces cerevisiae*	P03870.1	*Flipases*
CAA39900.1	*Lachancea waltii*	CAA39900.1	*Flipases*
ACY30629.1	*Zygosaccharomyces rouxii*	ACY30629.1	*Flipases*
SJM84337.1	*Zygosaccharomyces bailii*	SJM84337.1	*Flipases*
YP_006560839.1	*Salmonella phage vB_SosS_Oslo*	YP_006560839.1	*Phage IN*
YP_009219097.1	*Mycobacterium phage Anubis*	YP_009219097.1	*Phage IN*
BPMFR	*Mycobacterium phage FRAT1*	P25426.1	*Phage IN*
YP_008240364.1	*Thermus phage phi OH2*	YP_008240364.1	*Phage IN*
ANJ65425.1	*Lactobacillus phage PLE3*	ANJ65425.1	*Phage IN*
YP_009224245.1	*Erysipelothrix phage SE-1*	YP_009224245.1	*Phage IN*
AEV89266.1	*Bacillus phage phIS3501*	AEV89266.1	*Phage IN*
AXQ64381.1	*Gordonia phage Neville*	AXQ64381.1	*Phage IN*
BP186	*Escherichia virus 186*	P06723.1	*Phage IN*
BPHP1	*Haemophilus phage*	P21422.1	*Phage IN*
BPP2	*Escherichia virus P2*	P36932.2	*Phage IN*
YP_007237580.1	*Cronobacter phage ENT47670*	YP_007237580.1	*Phage IN*
BPHK0	*Escherichia virus HK022*	P16407.1	*Phage IN*
AAA67900.1	*Phage 434*	AAA67900.1	*Phage IN*
Lambda	*Escherichia virus HK022*	NP_037686.1	*Phage IN*
AAC48894.1	*Phage 21*	AAC48894.1	*Phage IN*
WP_006248153.1	*Mannheimia haemolytica*	WP_006248153.1	*Prok.Transposon*
YF72_HAEIN	*Haemophilus influenzae*	P46495.2	*Prok.Transposon*
RCl2_ECOLI	*Escherichia coli*	P16470.1	*Prok.Transposon*
ESQ72044.1	*Pasteurella multocida subsp.*	ESQ72044.1	*Prok.Transposon*
WP_039310837.1	*Pectobacterium atrosepticum*	WP_039310837.1	*Prok.Transposon*
EPO60639.1	*Klebsiella pneumoniae UHKPC17*	EPO60639.1	*Prok.Transposon*
WP_096215753.1	*Enterobacter hormaechei*	WP_096215753.1	*Prok.Transposon*
XERC_ECOLI	*Escherichia coli*	P22885	*Resolvase*
XERC_KSAC	*Kosakonia sacchari*	WP_065369792.1	*Resolvase*
XERC_EMALL	*Erwinia mallotivora*	WP_034938879.1	*Resolvase*
XERC_DZEAE	*Dickeya zeae*	WP_023640960.1	*Resolvase*
XERC_PCARO	*Pectobacterium carotovorum*	WP_039474195.1	*Resolvase*
XERD_ECOLI	*Escherichia coli*	P0A8P8.1	*Resolvase*
XERD_HAEIN	*Haemophilus influenzae*	L42023.1	*Resolvase*
XERD_CORIS	*Chelonobacter oris*	WP_034614898.1	*Resolvase*
XERD_VPSI	*Volucribacter psittacicida*	WP_132689482.1	*Resolvase*
XERD_XINN	*Xenorhabdus innexi*	SIP72660.1	*Resolvase*
DIRS1_CCri	*Chondrus crispus*	Repbase	*PAT-like*
Kangaroo	*Volvox carteri f. nagariensis*	AAM94957.1	*PAT-like*
DIRS1_NGr	*Naegleria gruberi*	Repbase	*PAT-like*
SkowPat	*Saccoglossus kowalevskii*	ACQM01123180	*PAT-like*
DIRS1_SK	*Saccoglossus kowalevskii*	Repbase	*PAT-like*
DIRS1_CGi	*Crassostrea gigas*	Repbase	*PAT-like*
HchiNgaro	*Hynobius chinensis*	GAQK01079872	*Ngaro-like*

LvNgaro1	*Lytechinus variegatus*	BK001253	*Ngaro-like*
DIRS7_CGi	*Crassostrea gigas*	Repbase	*Ngaro-like*
SpNgaro3	*Strongylocentrotus purpuratus*	AAGJ04006304	*Ngaro-like*
DIRS32_ACar	*Anolis carolinensis*	Repbase	*Ngaro-like*
Ngaro1_DR	*Danio rerio*	Repbase	*Ngaro-like*
LvNgaro2	*Lytechinus variegatus*	AGCV01398517	*Ngaro-like*
XtNgaro2	*Xenopus tropicalis*	AC175582	*Ngaro-like*
DIRS22_NV	*Nematostella vectensis*	Repbase	*Ngaro-like*
DIRS6_CGi	*Crassostrea gigas*	Repbase	*Ngaro-like*
DIRS1	*Dictyostelium discoideum*	M11339.1	*DIRS-like*
DIRS2_DPu	*Daphnia pulex*	Repbase	*DIRS-like*
DIRS8_NV	*Nematostella vectensis*	Repbase	*DIRS-like*
DIRS8_Lch	*Latimeria chalumnae*	Repbase	*DIRS-like*
DIRS1_BF	*Branchiostoma floridae*	Repbase	*DIRS-like*
DIRS1_NV	*Nematostella vectensis*	Repbase	*DIRS-like*
DIRS1_NVi	*Nasonia vitripennis*	Repbase	*DIRS-like*
DIRSNVi	*Nasonia vitripennis*	Repbase	*DIRS-like*
DIRS1_SIn	*Solenopsis invicta*	Repbase	*DIRS-like*
DIRS1_PBa	*Pogonomyrmex barbatus*	Repbase	*DIRS-like*
DIRS1_PH	*Parhyale hawaiensis*	Repbase	*DIRS-like*
EvenDirs	*Echinogammarus veneris*	GARO01000003	*DIRS-like*
DIRS1_LGI	*Lottia gigantea*	Repbase	*DIRS-like*
DIRS1_CTe	*Capitella teleta*	Repbase	*DIRS-like*
DIRS7_XL	*Xenopus laevis*	Repbase	*DIRS-like*
DIRS8_PSi	*Pelodiscus sinensis*	Repbase	*DIRS-like*
DIRS6_CPB	*Chrysemys picta bellii*	Repbase	*DIRS-like*
DIRS1_ACar	*Anolis carolinensis*	Repbase	*DIRS-like*
DIRS3_XT	*Xenopus tropicalis*	Repbase	*DIRS-like*
DIRS12_DR	*Danio rerio*	Repbase	*DIRS-like*
OAA29255.1	*Frankia sp. EI5c*	OAA29255.1	*Uncharacacterized YR*
WP_095512658.1	*Rubrivirga marina*	WP_095512658.1	*Uncharacacterized YR*
WP_083743821.1	*Methylobacterium radiotolerans*	WP_083743821.1	*Uncharacacterized YR*
WP_081839154.1	*Thermogemmatispora*	WP_081839154.1	*Uncharacacterized YR*
WP_043837840.1	*Roseomonas aerilata*	WP_043837840.1	*Uncharacacterized YR*
WP_079604047.1	*Bradyrhizobium erythrophlei*	WP_079604047.1	*Uncharacacterized YR*
RRA50387.1	*Acidipila sp. EB88*	RRA50387.1	*Uncharacacterized YR*
PCI44549.1	*Alphaproteobacteria bacterium*	PCI44549.1	*Uncharacacterized YR*
PZU19294.1	*Shinella sp.*	PZU19294.1	*Uncharacacterized YR*
WP_081629418.1	*Agrobacterium*	WP_081629418.1	*Uncharacacterized YR*
WP_037487218.1	*Sphingomonas paucimobilis*	WP_037487218.1	*Uncharacacterized YR*
WP_107804493.1	*Nitrosomonas oligotropha*	WP_107804493.1	*Uncharacacterized YR*
WP_074693775.1	*Alicyclobacillus hesperidum*	WP_074693775.1	*Uncharacacterized YR*
PJD94563.1	*Parachlamydia sp.*	PJD94563.1	*Uncharacacterized YR*
WP_090932552.1	*Pelosinus propionicus*	WP_090932552.1	*Uncharacacterized YR*
WP_091569285.1	*Melghirimyces thermohalophilus*	WP_091569285.1	*Uncharacacterized YR*
WP_105410419.1	*Paenibacillus sp. PCH8*	WP_105410419.1	*Uncharacacterized YR*
ALG42622.1	*Megasphaera elsdenii 14-14*	ALG42622.1	*Uncharacacterized YR*
PDH19566.1	*Pelagibacterales bacterium MED-*	PDH19566.1	*Uncharacacterized YR*
WP_047186324.1	*unclassified Bacilli*	WP_047186324.1	*Uncharacacterized YR*

WP_106215579.1	*Kineococcus rhizosphaerae*	WP_106215579.1	*Uncharacacterized YR*
WP_104433015.1	*Kineococcus xinjiangensis*	WP_104433015.1	*Uncharacacterized YR*
CryptonA-1_DR	*Danio rerio*	Repbase	*Crypton*
CryptonA-2_SP	*Strongylocentrotus purpuratus*	Repbase	*Crypton*
CryptonS-10_PI	*Phytophthora infestans*	Repbase	*Crypton*
CryptonA-1_SK	*Saccoglossus kowalevskii*	Repbase	*Crypton*
CryptonV-1_NV	*Nematostella vectensis*	Repbase	*Crypton*
CryptonS-2_PU	*Pythium ultimum*	Repbase	*Crypton*
CryptonS-1_PR	*Phytophthora ramorum*	Repbase	*Crypton*
CryptonI-1_RPro	*Rhodnius prolixus*	Repbase	*Crypton*
CryptonI-1_CQ	*Culex quinquefasciatus*	Repbase	*Crypton*
CryptonI-1_AA	*Aedes aegypti*	Repbase	*Crypton*
XerA	*Pyrococcus furiosus*	WP_011013007.1	XerA
IntI	*Shigella sonnei*	AAT72891.1	IntI
SNJ2	*Haloferax volcanii DS2*	ADE02447.1	SNJ2
IntC	*Yersinia enterocolitica*	WP_011817054.1	IntC
PhiChi	*Halovirus HCTV-5*	YP_008059154.1	PhiChi
pTN3	*Pyrococcus sp. NA2*	WP_013748767.1	pTN3
SSV1	*Sulfolobus virus Ragged Hills*	NP_963933.1	SSV1
PNOB8	*Sulfurisphaera tokodaii*	WP_010979387.1	PNOB8
IntG	*Epibacterium mobile*	WP_065323774.1	IntG
DAI	*Neisseria gonorrhoeae*	WP_082277758.1	DAI
Shuflon	*Salmonella enterica*	WP_050303304.1	Shuflon
CRE	*Escherichia virus P1*	YP_006472.1	CRE
BJ1	*Halobellus rufus*	WP_049986559.1	BJ1
